# Genomic Loci Affecting Milk Production in German Black Pied Cattle (DSN)

**DOI:** 10.3389/fgene.2021.640039

**Published:** 2021-03-08

**Authors:** Paula Korkuć, Danny Arends, Katharina May, Sven König, Gudrun A. Brockmann

**Affiliations:** ^1^Albrecht Daniel Thaer-Institute for Agricultural and Horticultural Sciences, Animal Breeding Biology and Molecular Genetics, Humboldt University Berlin, Berlin, Germany; ^2^Institute of Animal Breeding and Genetics, Justus-Liebig-University of Giessen, Giessen, Germany

**Keywords:** genome-wide association study, cattle, SNP chip, Holstein cattle, CSN3 gene, DGAT1 gene, casein gene

## Abstract

German Black Pied cattle (DSN) is an endangered population of about 2,550 dual-purpose cattle in Germany. Having a milk yield of about 2,500 kg less than the predominant dairy breed Holstein, the preservation of DSN is supported by the German government and the EU. The identification of the genomic loci affecting milk production in DSN can provide a basis for selection decisions for genetic improvement of DSN in order to increase market chances through the improvement of milk yield. A genome-wide association analysis of 30 milk traits was conducted in different lactation periods and numbers. Association using multiple linear regression models in R was performed on 1,490 DSN cattle genotyped with BovineSNP50 SNP-chip. 41 significant and 20 suggestive SNPs affecting milk production traits in DSN were identified, as well as 15 additional SNPs for protein content which are less reliable due to high inflation. The most significant effects on milk yield in DSN were detected on chromosomes 1, 6, and 20. The region on chromosome 6 was located nearby the casein gene cluster and the corresponding haplotype overlapped the *CSN3* gene (casein kappa). Associations for fat and protein yield and content were also detected. High correlation between traits of the same lactation period or number led to some SNPs being significant for multiple investigated traits. Half of all identified SNPs have been reported in other studies, previously. 15 SNPs were associated with the same traits in other breeds. The other associated SNPs have been reported previously for traits such as exterior, health, meat and carcass, production, and reproduction traits. No association could be detected between *DGAT1* and other known milk genes with milk production traits despite the close relationship between DSN and Holstein. The results of this study confirmed that many SNPs identified in other breeds as associated with milk traits also affect milk traits in dual-purpose DSN cattle and can be used for further genetic analysis to identify genes and causal variants that affect milk production in DSN cattle.

## Introduction

German Black Pied cattle (DSN, “Deutsches Schwarzbuntes Niederungsrind”) is an endangered breed of about 2,550 dual-purpose cattle in Germany. The ancestor population of this breed is considered as one of the founder populations of the nowadays dominantly used high yielding German Holstein breed (Köppe-Forsthoff, 1967; Grothe, 1993), which is one reason why the German government and the European Union support its preservation. This support is necessary because milk yield of DSN cows is about 2,500 kg less compared to German Holstein, which has led to the replacement of DSN by Holstein cattle. Although DSN is a dual-purpose breed, milk yield is the main contributor to the economic merit and meat yield and carcass quality do not compensate the lower milk yield.

The interest in preservation of DSN does not only stem from its relationship to Holstein cattle and retaining genetic diversity as a gene reserve for the future. The interest in DSN cattle is also due to their advantageous traits. For example, the milk fat and protein content with 4.3 % and 3.7 %, respectively, is higher compared to German Holstein (RBB Rinderproduktion Berlin-Brandenburg GmbH., 2020). Moreover, DSN cattle are considered to be more robust for grazing and more fertile.

One of the long-term goals for maintaining DSN is to reduce governmental financial support by increasing economic value of the breed through the improvement of milk yield. Simultaneously, the advantageous traits and the typical body composition of this dual-purpose breed should be maintained.

So far, little is known about the genes affecting milk traits in DSN. Recently, genome-wide association studies (GWAS) in DSN for health traits identified three significant and two suggestive SNPs for clinical mastitis (Meier et al., 2020) and 44 significant SNPs for endoparasite resistance (May et al., 2019). Another study, investigating genomic variation in the casein gene cluster, found three protein variants of CSN2 and CSN3 and fixed protein variants for CSN1S1 and CSN1S2 (Meier et al., 2019). In contrast, 63,404 associations with milk traits in general were available from Cattle QTLdb Release 42 (accessed 09/21/2020) (Hu et al., 2019), whereof around 79 % were found in studies with Holstein cattle. Furthermore, 10 % of the reported associations in those studies with Holstein cattle are reported within the first 10 Mb on chromosome 14, where the *DGAT1* gene is located which is known to influence milk yield and composition (Grisart et al., 2002).

In this study, we investigated the genetic basis contributing to the variation in milk performance of the current DSN population by GWAS. Since the DSN population is small, power to find significant genomic loci is limited. Nevertheless, there is an urgent need to provide genetic association information for small endangered populations to support their preservation and further development. Even if not all genomic loci are significantly detectable, the obtained results in DSN and the comparison to related breeds provide a basis for selection decisions to genetically improve DSN.

## Materials and Methods

### Populations

Data of 1,816 DSN cows was available for this study. These cows represent about two thirds of the DSN population registered in Germany (The Society for the Conservation of Old and Endangered Livestock Breeds (GEH), 2018). The cows were born between 2005 and 2016, and descended from 76 sires. Cows were raised on six farms. In order to reduce environmental influences, we filtered to have at least 20 DSN cows per farm, per sire, and per birth year. This reduced the data set to 1,490 DSN cows from five farms, born between 2007 and 2016, and descending from 28 sires.

In order to compare the results that we obtained in DSN with German Holstein, we used GWAS results previously obtained in our lab on a population of 2,400 German Holstein bulls. This population has been used and described in detail repeatedly (Zielke et al., 2011, 2013; Abdel-Shafy et al., 2018).

### Phenotypes

Milk traits with corresponding pedigree data were obtained from the cattle breeding association “RBB Rinderproduktion Berlin-Brandenburg GmbH” in April 2020. Traits included milk, fat, and protein yield in kilogram (milk kg, fat kg, and protein kg) for three lactation periods: 100-days (100d), 200-days (200d), and 305-days (305d). 305 days data was available for the first three lactations (LA1, LA2, and LA3), whereas 100 and 200 days data was available only for LA1. 305 days data of cows with < 270 days in milk was not considered. Fat and protein content (fat %, protein %) were calculated by dividing fat or protein kg by milk kg of the respective lactation. The lactation mean (LAm) was calculated for cows with full data in the first three lactations. This leads to a total of 30 investigated milk traits in this study. For each trait, outliers were defined as values outside the 1.5 times interquartile range within each farm and removed from the data set. This leads to data being available for 1,478, 1,476, 1,372, 1,160, 862, and 685 DSN cows in LA1 (100d), LA1 (200d), LA1, LA2, LA3, and LAm, respectively.

### Genotypes

DSN cattle cows were genotyped using the Illumina^®^ Bovine50SNP v3 BeadChip (Illumina, Inc., 5200 Illumina Way, San Diego, CA, United States). SNP chip probe sequences were remapped against the *Bos taurus* genome version ARS-UCD1.2 (Rosen et al., 2018) using NCBI Nucleotide-Nucleotide BLAST version 2.2.31+ (Altschul et al., 1990) in order to obtain genome positions for SNPs on the ARS-UCD1.2 genome build. SNP probes that mapped to multiple genomic locations were removed. Genotype quality control was performed for animals and SNPs. SNP calls with a GC-score < 0.7 were set to missing. Animals with a call rate < 90% were discarded. SNPs with a call rate < 95% and a minor allele frequency (MAF) < 5% were removed. Lastly, genotype groups with less than 30 observations were set to missing to prevent spurious association. After quality control, 36,929 high confident SNPs were available for further analysis.

### Genome-Wide Association Study

Genome-wide association studies was performed with multiple linear regression models implemented in the R language for statistical computing (version 4.0.3) (R Core Team, 2018). Various models were tested and compared by calculating the inflation factor λ of each model to judge the extent of the excess of type-I errors (Devlin and Roeder, 1999). Finally, the model with the lowest λ was selected (Supplementary Table 1). On average 27 % of top 100 SNPs of the selected model are shared with each of the other models tested (Supplementary Figure 1). The resulting model for testing the additive effect of each SNP was:

Yi⁢j⁢k⁢l⁢m⁢n⁢o⁢p⁢q=p⁢si+fj*+⁢sk*+⁢b⁢yl*⁢+b⁢sm*⁢+c⁢yn*

(1)   +⁢c⁢so*+⁢a⁢cp*⁢+gq+ei⁢j⁢k⁢l⁢m⁢n⁢o⁢p⁢q

where *Y*_*ijklmnopq*_ is the trait, *ps*_*i*_ represents the covariate for population stratification followed by the covariates for farm *f*_*j*_, sire *s*_*k*_, birth year *by*_*l*_, birth season *bs*_*m*_, calving year *cy*_*n*_, calving season *cs*_*o*_, age at first calving in days *ac*_*p*_, the SNP genotype *g*_*q*_, and *e*_*ijklmnopq*_ is the residual error. Covariates marked with an asterisk “^∗^” were only included into the model when the difference in the Akaike information criterion (ΔAIC) was ≤ −10 between the null model (*Y*_*i*_=*ps*_*i*_) and the null model extended with one of the covariates (*Y*_*ix*_=*ps*_*i*_+*covariate*_*x*_) (Supplementary Table 2). All covariates were included as fixed effects as this resulted in the lowest inflation factor λ. Population stratification *ps*_*i*_ among the 1,490 DSN cows was examined using pairwise population concordance tests on an identity-by-state matrix implemented in PLINK (version 1.90) with a *p*-value cut-off of 0.0001 (Purcell et al., 2007; Chang et al., 2015). The resulting 33 clusters of relatedness were included as a covariate in the GWAS model.

Interactions between lactation stages and SNP genotypes were investigated for 100, 200, and 305 days performance data in LA1 and between lactation number and SNP genotypes for 305 days performance data in lactations 1 to 3. For testing the genetic interaction effects between milk, fat, and protein yield data and specific lactation periods, the difference between the difference between birth and 100 days, 100 and 200 days, and 200 and 305 days performance data was used as input data. A linear mixed-effects model as described by Lu and Bovenhuis (2019) was fitted using the R package lmerTest (version 3.1-3) (Kuznetsova et al., 2017):

$$Y_{i\,j\,k\,l\,m\,n\,o\,p\,q\,r\,s}=p\,s_{i}+f_{j}^{*}+\,s_{k}^{*}+\,b\,y_{l}^{*}+\,b\,s_{m}^{*}+\,c\,y_{n}^{*}$$

(2) $$+\,c\,s_{o}^{*}+\,a\,c_{p}^{*}+g_{q}+L_{r}+g_{q}\,\,x\,\,L_{r}+\left(1|a\,n\,i\,m\,a\,l_{s}\right)+e_{i\,j\,k\,l\,m\,n\,o\,p\,q\,r\,s}$$

where the same covariates were used as in Eq. 1 but extended by the lactation stage or lactation number *L*_*r*_, the interaction term between lactation stage or lactation number and SNP genotype *L_*r*_ x g_*q*_*, and a random intercept included for each individual animal *(1| animal_*s*_)* to compensate for repeated measurements on the animals. Covariates marked with an asterisk “^∗^” were only included into the model when the difference in the Akaike information criterion (ΔAIC) was ≤ = −10 between the null model [*Y*_*s*_=(1|*animal*_*s*_)] and the null model extended with one of the covariates [*Y*_*xs*_=*covariate*_*x*_+(1|*animal*_*s*_)] (Supplementary Table 3). Separate analyses were performed for the traits milk, fat and protein yield, and fat and protein content.

The significance threshold was adjusted for multiple testing using Bonferroni (BF) correction. The number of independent tests (*M*_*eff*_) was estimated by the simpleM method, to account for linkage between neighboring SNPs (Gao et al., 2008). Window sizes from 100 to 630 (630 corresponds to the minimum number of SNPs in one out of all chromosomes) in this study were tested. The lowest estimated *M*_*eff*_ value was 18,278 at a window size of 630 which was then used for Bonferroni correction. After Bonferroni correction, SNPs were considered highly significant when P_*BF*_ < 0.01, significant when P_*BF*_ < 0.05, or suggestive when P_*BF*_ < 0.1. In the case of interaction terms, *M*_*eff*_ was multiplied by the number of lactation stages or lactation numbers (*n* = 18,278^∗^3) and *p*-values were noted as P_*BI*_. Figures were produced using the R package ggplot2 (version 3.3.2) (Wickham et al., 2016) unless otherwise stated. For SNP effect plots, *p*-values between genotype groups were estimated using pairwise *t*-tests and displayed using R package ggpubr (version 0.4.0) (Kassambara, 2020).

### Haplotypes and Gene Annotation

Haplotype blocks in which SNPs are located were computed using Haploview version 4.2 (Barrett et al., 2005). In order to define blocks in Haploview, the customized block definition was used with the option “solid LD spine” set to D′ > 0.6. Genes within each haplotype were annotated using R package biomaRt (version 2.48.0) (Durinck et al., 2009) using the Ensembl *Bos taurus* database based on the ARS-UCD1.2 assembly (Yates et al., 2020). If no haplotype block could be estimated for a SNP, genes within ± 70 kb up- and down-stream (corresponding to the median haplotype block length of 140 kb) of the respective SNP position were considered as positional candidate genes. Additionally, the 1 Mb region centered around the investigated SNP was inspected for candidate genes. If consecutive 1 Mb regions overlapped, they were merged by taking the start of the first and the end of the second region.

### Overlap With Cattle QTLdb

Associated SNPs were compared to GWAS results and known QTLs from Cattle QTLdb Release 42 (Hu et al., 2019) by using their SNP-IDs (rs). Further, also the 1 Mb region centered around the associated SNP was used to find overlapping loci with other publications. The entries from Cattle QTLdb were categorized into the trait categories “exterior,” “health,” “meat and carcass,” “milk,” “production,” and “reproduction.”

## Results

### Associations for Almost All Traits

Genome-wide association analyses using 1,490 DSN cows and 36,929 high confident SNPs identified associations for all traits except for 100-days fat yield in lactation 1 (fat kg 100 days LA1) and the lactation mean of the 305-days fat yield (fat kg LAm). The inflation factor λ ranged from 1.27 for fat yield (LA2 305 days) to 2.19 for protein content (LA2 305 days) although extensive efforts were taken to select a GWAS model that showed the lowest overall inflation (Supplementary Table 1). Nonetheless, the inflation factor λ and the Q-Q-plots for all traits (Supplementary Figure 2) showed that the level of statistical significance was overestimated in general. We assume that this especially holds for protein content in all lactation periods and numbers and for fat content in LA1 (100 and 305 days). The number of false positives was increased as λ-values higher or equal to 1.5 were observed for these traits. Even if we reduce the genome-wide significance threshold to *P*_*BF*_ < 0.0005, 16 SNPs remain associated. The results of those traits are listed in the Supplementary Table 4. Results of associations for 20 traits where λ was below 1.5 are presented in Table 1. For those traits, 14 highly significant, 27 significant, and 20 suggestive SNPs were found. Some SNPs were associated with multiple traits because of high correlation between those traits (Supplementary Tables 5,6). High correlation was found between performance data within the first lactation (100, 200, and 305 days of LA1) (*r* > 0.76), and between traits of the first three lactations (LA1, LA2, and LA3) and the lactation mean (LAm) (*r* > 0.77). Fat and protein yield within a lactation were also highly correlated (*r* > 0.75).

**TABLE 1:** GWAS for milk production traits in DSN.

**Chr**	**Position (bp)**	**SNP-ID**	**Ref**	**Alt**	**MA**	**MAF**	**Trait**	***N***	**β**	**SE(β)**	**P_*BF*_**
1	77,049,090	rs110516247	A	C	C	0.11	Milk kg (LA3)	846	−515	100	**0.00679**
							Protein kg (LA3)	844	−16.3	3.3	0.02351
	79,757,250	rs43246393	C	T	T	0.34	Milk kg (LA3)	856	−390	76	**0.00020**
							Protein kg (LA3)	854	−13.2	2.5	**0.00007**
							Protein kg (LAm)	679	−10.3	2.2	0.02641
							Fat kg (LA3)	857	−13.4	3.1	0.01868
	115,539,332	rs42347234	C	T	T	0.11	Milk kg (LA1)	1355	336	70	0.03846
							Protein kg (LA1)	1357	11.9	2.4	0.01140
	128,503,005	rs109686415	C	T	C	0.49	Fat % (LA2)	1115	−0.082	0.016	**0.00357**
							Fat % (LAm)	660	−0.082	0.016	0.01599
	143,812,919	rs41255272	A	G	A	0.50	Milk kg (LA1)	1367	204	41	0.01478
							Protein kg (LA1)	1369	6.6	1.4	0.04613
2	123,100,345	rs110278850	A	C	C	0.45	Protein kg (200 days)	1436	3.5	0.8	0.04941
3	15,947,663	rs110565504	T	C	T	0.24	Fat % (200 days)	1458	0.056	0.019	0.03398
4	47,674,986	rs110143001	G	A	G	0.42	Fat % (LA3)	844	0.086	0.019	0.04275
	91,071,208	rs42753220	C	T	C	0.40	Milk kg (LA1)	1370	213	44	0.01390
5	62,094,191	rs29009717	A	G	A	0.18	Fat % (LA3)	853	−0.125	0.032	0.09655
	83,959,138	rs41660560	G	T	G	0.35	Protein kg (LA2)	1160	9.4	2.1	0.01767
	93,953,629	rs109945272	T	C	T	0.24	Fat % (LA2)	1136	−0.077	0.024	0.08780
	114,935,739	rs41652414	C	T	T	0.19	Milk kg (LA1)	1357	220	71	0.02814
							Milk kg (100 days)	1457	64	24	0.08596
							Milk kg (200 days)	1452	141	46	**0.00923**
							Protein kg (LA1)	1359	7.2	2.4	0.04450
							Protein kg (100 days)	1459	2	0.7	0.08697
							Protein kg (200 days)	1457	4.1	1.4	0.02288
							Fat kg (200 days)	1447	5.1	1.7	0.07535
6	60,162,206	rs41605188	G	A	A	0.34	Milk kg (200 days)	1469	119	29	0.09377
	61,733,082	rs135571989	C	A	A	0.21	Milk kg (LA1)	1320	−195	71	0.07717
							Milk kg (200 days)	1416	−110	45	0.08954
	62,988,117	rs42436495	T	G	G	0.30	Milk kg (LA1)	1370	225	49	0.01789
							Milk kg (200 days)	1469	136	32	0.03921
	63,010,380	rs42436482	A	G	G	0.29	Milk kg (LA1)	1368	220	52	0.04884
							Milk kg (200 days)	1467	132	34	0.09298
	64,928,624	rs42482917	T	C	C	0.32	Milk kg (200 days)	1463	111	30	0.09593
	77,688,509	rs41652041	A	G	G	0.35	Milk kg (LA1)	1372	−212	49	0.06866
							Milk kg (200 days)	1470	−138	31	0.03904
	80,530,130	rs110291935	T	C	T	0.41	Milk kg (LA1)	1370	−209	42	0.01362
							Milk kg (100 days)	1474	−71	14	**0.00519**
							Milk kg (200 days)	1469	−132	27	**0.00655**
	80,626,467	rs109872424	T	C	T	0.33	Milk kg (100 days)	1473	−64	16	0.06727
							Milk kg (200 days)	1468	−124	30	0.02416
	86,112,142	rs109592101	A	G	A	0.42	Milk kg (LA1)	1372	−193	41	0.01990
							Milk kg (100 days)	1476	−78	14	**0.00009**
							Milk kg (200 days)	1471	−119	26	0.04162
							Protein kg (100 days)	1478	−1.8	0.4	0.08431
	87,266,808	rs41591365	C	T	C	0.46	Protein kg (100 days)	1473	2.1	0.4	0.03608
	88,164,411	rs41622837	A	G	A	0.14	Protein kg (200 days)	1444	−6	1.3	0.07033
8	53,663,120	rs41793393	C	T	T	0.19	Milk kg (LA3)	847	−356	115	**0.00508**
							Milk kg (LAm)	673	−316	97	0.03554
	53,867,972	rs109542652	G	A	A	0.14	Milk kg (LA3)	846	−489	101	0.02538
	59,101,606	rs43550935	A	G	G	0.19	Milk kg (LAm)	685	−271	95	0.07475
							Protein kg (LAm)	685	−9.1	3.2	0.02661
	100,876,785	rs108983661	G	A	A	0.25	Fat kg (LAm)	683	12.5	3	0.05697
9	10,638,013	rs133869947	A	C	A	0.38	Milk kg (LA3)	818	−297	70	0.06280
							Milk kg (LAm)	645	−270	60	0.04397
	26,353,699	rs110314239	G	T	T	0.48	Fat kg (LA1)	1366	−6.8	1.5	0.05814
10	3,611,602	rs42697353	T	C	C	0.49	Fat % (200 days)	1449	−0.061	0.013	0.03506
11	69,443,503	rs110564084	T	C	T	0.30	Protein kg (LA2)	1157	11.9	2.5	0.05681
	92,712,210	rs110540697	T	C	C	0.41	Fat % (200 days)	1454	0.057	0.013	0.09715
12	66,340,756	rs41629344	T	G	G	0.39	Fat % (200 days)	1452	0.06	0.013	**0.00380**
14	26,340,400	rs41727315	G	A	A	0.06	Protein kg (100 days)	1470	4	0.9	0.06447
16	11,516,923	rs41623175	A	G	A	0.21	Fat % (200 days)	1459	−0.082	0.02	0.05676
	30,668,830	rs43041491	G	T	G	0.11	Fat % (LA3)	835	0.153	0.031	0.02338
	32,105,683	rs798259422	A	G	G	0.29	Fat % (200 days)	1458	−0.055	0.015	0.05996
	33,789,714	rs41796289	C	T	C	0.39	Fat % (LA2)	1134	−0.061	0.015	0.02776
							Fat % (200 days)	1450	−0.049	0.013	0.09433
	40,391,486	rs41804404	A	G	G	0.46	Fat % (200 days)	1446	0.058	0.012	0.04642
	46,683,276	rs110777881	A	G	G	0.36	Protein kg (100 days)	1444	−1.8	0.5	0.05105
	46,773,225	rs43719805	T	C	C	0.47	Protein kg (100 days)	1472	1.9	0.4	0.04084
18	53,596,284	rs109907036	C	T	T	0.37	Fat % (LA2)	1136	0.086	0.017	**0.00477**
20	50,879,180	rs41948928	T	C	T	0.10	Fat % (200 days)	1445	−0.118	0.023	**0.00777**
	71,448,297	rs110353352	C	T	C	0.13	Milk kg (LA2)	1137	484	86	**0.00040**
							Protein kg (LA2)	1143	15	2.9	**0.00503**
							Fat kg (LA2)	1137	17.3	3.5	0.01434
21	42,828,439	rs41978846	T	G	T	0.33	Protein kg (200 days)	1450	−4.2	0.9	0.09136
24	16,789,760	rs110860585	C	T	T	0.41	Milk kg (LA3)	862	−348	72	0.01192
	30,250,034	rs110476141	G	A	G	0.36	Protein kg (LA1)	1355	−7	1.5	0.01207
							Protein kg (200 days)	1453	−4.1	0.9	0.03475
							Fat kg (LA1)	1348	−8.5	1.7	0.02349
25	7,944,597	rs109583598	T	C	C	0.29	Fat % (200 days)	1456	0.065	0.016	0.01082
	9,711,895	rs110469759	A	G	A	0.17	Fat kg (LA1)	1368	10.8	3.5	**0.00392**
							Fat kg (200 days)	1467	5.7	2	0.02959
	1,1019,450	rs109027867	G	T	G	0.48	Milk kg (200 days)	1445	−128	26	0.03396
27	13,864,569	rs110009442	C	G	C	0.32	Fat % (LA2)	1125	0.061	0.018	0.08067
							Fat % (200 days)	1442	0.06	0.015	0.09215
	18,527,112	rs42957103	G	A	A	0.41	Fat kg (LA1)	1367	7.5	1.7	0.09536
28	25,196,334	rs41587054	A	G	G	0.10	Protein kg (100 days)	1419	3.8	0.8	0.06929
29	50,217,955	rs109241029	G	A	A	0.44	Fat % (LA2)	1137	0.076	0.016	0.04229
	50,260,533	rs109840529	A	G	G	0.42	Fat % (LA2)	1118	−0.082	0.016	**0.00338**
	50,326,170	rs110740589	A	G	A	0.41	Fat % (LA2)	1113	−0.09	0.016	**0.00022**
X	12,896,716	rs109188619	T	A	T	0.18	Milk kg (LA3)	860	546	122	0.02488
	116,886,837	rs41626783	T	C	C	0.38	Protein kg (200 days)	1476	−4.6	1.1	0.08409
	117,691,901	rs110011913	A	T	T	0.31	Protein kg (LA3)	851	−13.5	2.7	0.01321
							Protein kg (LAm)	676	−10.8	2.4	0.07065
	133,244,405	rs29018822	G	T	T	0.35	Fat kg (200 days)	1409	−6.8	1.4	0.07242

*For each trait, significantly associated SNPs are listed with chromosome number (Chr), chromosomal position in base pairs (bp), the SNP reference ID (SNP-ID), the given allele on the forward strand in the reference genome ARS-UCD1.2 (Ref), the alternative allele (Alt), the minor allele in the examined DSN population (MA), and the minor allele frequency (MAF). Furthermore, the associated trait, the number of cows in the specific analysis of this trait (n), the allele substitution effect of the minor allele (β), the standard error [SE(β)], and the *p*-value after Bonferroni-correction (P_*BF*_) are given. *P*-values of P_*BF*_ < 0.1, P_*BF*_ < 0.05, and P_*BF*_ < 0.01 were considered as suggestive (highlighted in gray), significant, or highly significant (highlighted in bold), respectively.*

### Effects on Milk Yield

**FIGURE 1:**
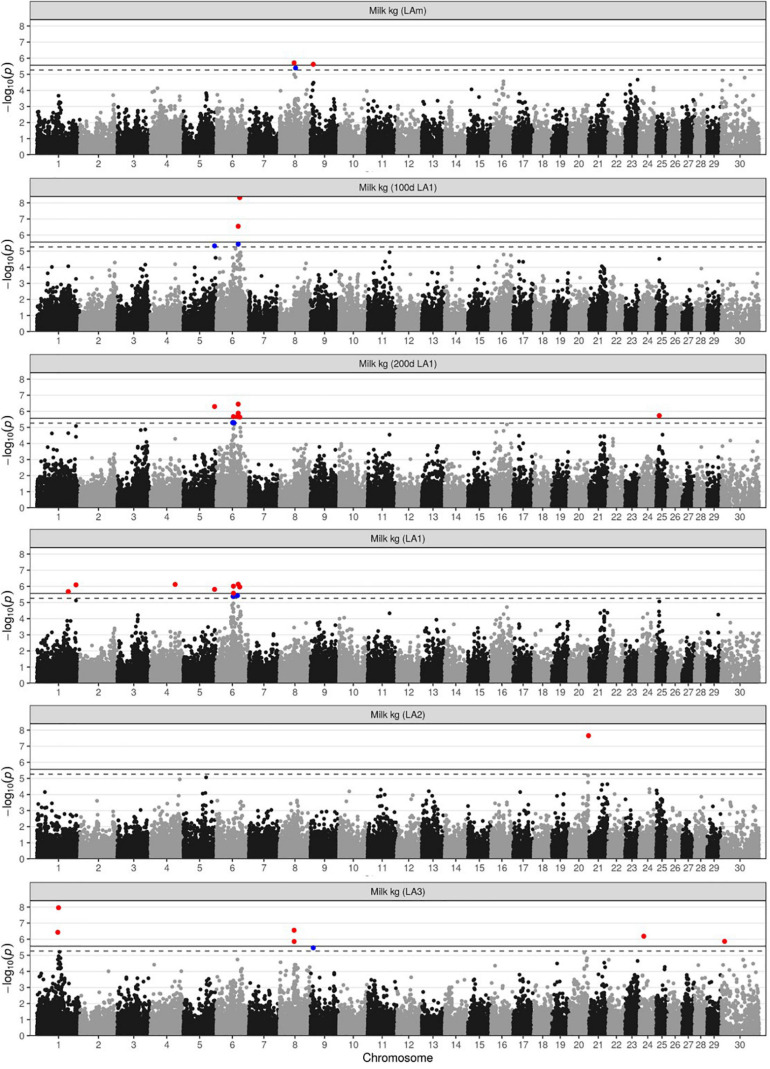
Manhattan plots for milk yield in kg. Plots are shown for the lactation mean of the 305 days performance (LAm), the 100 and 200 days performance in LA1, and the 305-days performance in the first three lactations (LA1-LA3). Markers above the significance or suggestive thresholds are highlighted in red (solid line, α < 0.05) or blue (dashed line, α < 0.1), respectively.

Since milk yield is the economically most important production trait, there is major interest in identifying genetic loci contributing to its variance in DSN cows (Table 1 and Figure 1). Using the mean milk yield across the first three lactations (LAm), only two loci were found to be associated, one on chromosome 8 at 53.7 Mb (8:53,663,120, rs41793393, P_*BF*_ = 0.03554) and another one on chromosome 9 at 10.6 Mb (9: 10,638,013, rs133869947, P_*BF*_ = 0.04397) (Supplementary Figures 3A,B). These two loci were also associated with milk yield in lactation 3 (LA3). Examining different lactation periods and lactations separately, we found additional loci on chromosomes 1, 4, 5, 6, 20, 24, 25, and X. Since the significance varied largely between lactation periods and lactation numbers, the interaction between the SNPs and lactation stage or lactation number was also investigated (Supplementary Table 7).

**FIGURE 2:**
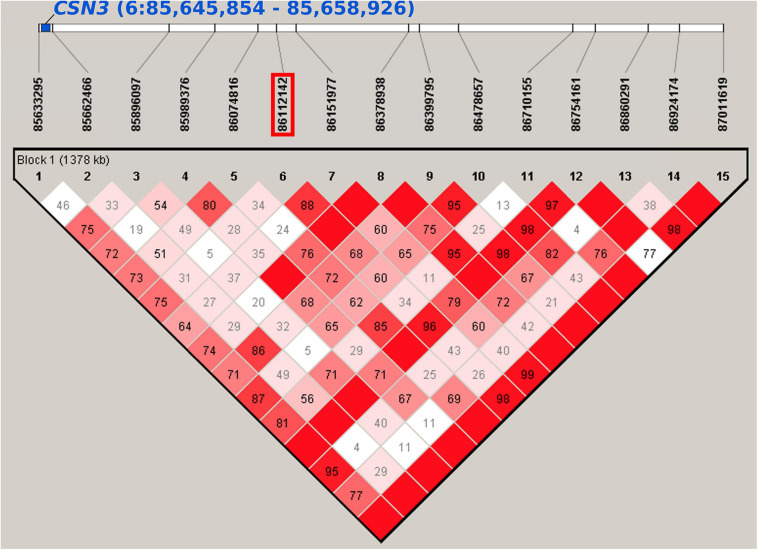
Haplotype block including SNP rs109592101 (6:86,112,142) estimated with Haploview. The investigated SNP is located in a haplotype block (6:85,633,295-87,011619) that overlaps with the position of the *CSN3* gene (6:85,645,854-85,658,926) which is known to be associated with milk production traits in several cattle breeds.

In the first lactation (LA1), chromosome 6 was most significantly associated (Table 1 and Figure 1). In the region between 60.2 and 87.2 Mb, 9 SNPs were associated with milk yield in all lactation periods of LA1. The top marker rs109592101 (6:86,112,142, *P_*BF*_* = 0.00009, Supplementary Figure 3C) showed the highest association with the 100 days performance. The minor allele A with a frequency of 0.42 accounted for a decrease in milk yield of 78 kg, 119 kg and 193 kg after 100, 200, and 305 days in lactation, respectively. The same SNP also suggestively affected (*P_*BF*_* = 0.08431) protein yield with a minor allele effect of −1.8 kg after 100 days in lactation. In the haplotype block around the top SNP (6:85,633,295-87,011,619, Figure 2 and Supplementary Table 8) 16 genes are located, among them *CSN3* (kappa casein) is known as main milk protein gene and as a gene affecting milk yield and composition in Holstein cattle (Mckenzie et al., 1984; Ng-Kwai-Hang et al., 1984). Another SNP in the same region on chromosome 6 (rs110291935, 6:80,530,130, *P_*BF*_* < 0.02, Supplementary Figure 3D) decreased milk yield until 100, 200, and 305 days in LA1 by 71, 132, and 209 kg, respectively. The corresponding haplotype block (6:80,530,130-80,626,467) did not contain any gene (Supplementary Table 8). However, the surrounding 1 Mb region harbors three genes ENSBTAG00000050977, *EPHA5* (EPH receptor A5), and ENSBTAG00000053125. Additional SNPs for milk yield in LA1 were found on chromosome 1 (rs42347234, 1:115,539,332, *P_*BF*_* = 0.03846; rs41255272, 1:143,812,919, *P_*BF*_* = 0.01478), chromosome 4 (rs42753220, 4:91,071,208, *P_*BF*_* = 0.01390), chromosome 5 (rs41652414, 5:114,935,739, *P_*BF*_* = 0.02814), and chromosome 25 (rs109027867, 25:11,019,450, *P_*BF*_* = 0.03396) (Supplementary Figures 3E–I). The minor allele of these SNPs showed an increase in milk yield by at least 64 kg, 141, and 204 kg for 100, 200, and 305 days performance data of LA1, respectively, expect for the SNP rs109027867 on chromosome 25 which showed a decrease of the minor allele by 128 kg for 200 days data.

In lactation 2 (LA2), the only association with milk yield was found on chromosome 20 (Table 1 and Figure 1) with the SNP rs110353352 (20:71,448,297, *P_*BF*_* = 0.00040, Supplementary Figure 3J). The minor allele C, which segregated at a frequency of 0.13, was advantageous for milk production increasing milk yield by 484 kg. This same SNP allele had also positive effects of 17.3 kg and 15.0 kg on fat and protein yield in LA2, respectively. The haplotype block of this SNP (20:71,378,297-71,518,297) contains five genes *TRIP13* (thyroid hormone receptor interactor 13), *BRD9* (bromodomain containing 9), *ENSBTAG00000054687*, *TPPP* (tubulin polymerization promoting protein), and *CEP72* (centrosomal protein 72) (Supplementary Table 8).

In lactation 3 (LA3), the most significant association for milk yield was found on chromosome 1 (Table 1 and Figure 1). The top SNP rs43246393 (1:79,757,250, *P_*BF*_* = 0.00020, Supplementary Figure 3K) was also associated with fat (*P_*BF*_* = 0.01868) and protein yield (*P_*BF*_* = 0.00007) in LA3 as well as with protein yield of the average across all three lactations (*P_*BF*_* = 0.02641). The minor allele T, segregating at a frequency of 0.34, accounted for 390 kg, 13.4 kg and 13.2 kg less milk, fat, and protein in LA3, respectively, and for 10.3 kg less protein in the lactation mean (LAm). The test for interaction between genotypes at this top SNP and lactation stage or lactation number showed significant effects on milk, fat, and protein yields (*P_*BI*_* < 0.03, Supplementary Table 7). The corresponding haplotype block around this SNP (1:79,606,618-79,757,250) contains three genes *BCL6* (BCL6 transcription repressor), *RTP2* (receptor transporter protein 2), and *SST* (somatostatin). In addition to the locus on chromosome 1, two SNPs on chromosome 8 were found to be associated (rs41793393, 8:53,663,120, *P_*BF*_* = 0.00508, Supplementary Figure 3l; rs109542652, 8:53,867,972, *P_*BF*_* = 0.02538). As mentioned above, this region was also identified for the average milk yield over lactations 1–3 (LAm). The minor allele T of the lead SNP rs41793393 (frequency 0.19) is decreasing milk yield in LA3 by 356 kg and in LAm by 316 kg. The corresponding haplotype block (8:53,467,317-54,449,954) contains the genes *GNA14* (G protein subunit alpha 14), *GNAQ* (G protein subunit alpha q), *CEP78* (centrosomal protein 78), and *PSAT1* (phosphoserine aminotransferase 1). Additional associations with milk yield in LA3 were found for SNP rs110860585 (24:16,789,760, *P_*BF*_* = 0.01192, Supplementary Figure 3M) and rs109188619 (X:12,896,716, *P_*BF*_* = 0.02488, Supplementary Figure 3N).

### Effects on Milk Fat Yield and Content

Regions associated with milk fat yield were identified on chromosomes 1, 20, 24, and 25 (Table 1 and Supplementary Figure 4). The most significant effect on fat yield was found on chromosome 25. The SNP rs110469759 (25:9,711,895, *P_*BF*_* = 0.00392, Supplementary Figure 3O) with an allele frequency of 0.17 showed an allele substitution effect of the A allele leading to an increase of 10.8 kg fat in LA1 and of 5.7 kg for 200 days performance in LA1. The association for fat yield on the other chromosomes (LA3: rs43246393, 1:79,757,250, *P_*BF*_* = 0.01868; LA2: rs110353352, 20:71,448,297, *P_*BF*_* = 0.01434; LA1: rs110476141, 24:30,250,034, *P*_*BF*_ = 0.02349, Supplementary Figures 3P–R) coincided with effects on milk and protein yield. The minor alleles on chromosomes 1 and 24 accounted for lower, the minor allele on chromosome 20 and 25 for higher milk, protein and fat yields (Table 1).

Highly significant SNPs associated with fat content were identified on chromosomes 1, 12, 18, 20, and 29 (Table 1 and Supplementary Figure 5). The most significant association was found on chromosome 29 with the top SNP rs110740589 (29:50,326,170, *P_*BF*_* = 0.00022, Supplementary Figure 3S). This SNP was associated with fat content in LA2. The minor allele A of this SNP (MAF = 0.41) showed a decrease in fat content in LA2 by 0.09% points. The neighboring SNP rs109840529 (29:50,260,533) showed interaction effects with the 100–305 days lactation stages of LA1 on fat content (*P_*BI*_* = 1.4E-07, Supplementary Table 7). On chromosome 1, the SNP rs109686415 (1:128,503,005) was associated with fat content in LA2 (*P_*BF*_* = 0.00357, Supplementary Figure 3T) and with the lactation mean of LA1-3 (*P_*BF*_* = 0.01599). This SNP was also significant for the interaction effect between SNP and lactation stages for the trait fat content across (*P_*BI*_* = 0.00016, Supplementary Table 7). The SNP rs109907036 on chromosome 18 (18:53,596,284, *P_*BF*_* = 0.00477, Supplementary Figure 3W) was also identified for fat content in LA2. The associations on chromosomes 12 (rs41629344, 12:66,340,756, *P_*BF*_* = 0.00380, Supplementary Figure 3U) and 20 (rs41948928, 20:50,879,180, *P_*BF*_* = 0.00777, Supplementary Figure 3V) were associated with fat content for 200 days performance data in LA1. The minor allele was the non-beneficial allele on chromosome 1 and 20, and the beneficial allele on chromosomes 12 and 18.

Additional loci affecting the changing fat contents during lactation 1 were found on chromosomes 5 and 6 (*p* < 0.0006, Supplementary Table 7). On chromosome 5 these were the SNPs rs41660560 (5:83,959,138, *P_*B*__*I*_* = 0.00003) and rs109945272 (5:93,953,629, *P_*B*__*I*_* = 1.9E-06) and on chromosome 6 the four SNPs rs41605188 (6:60,162,206, *P_*B*__*I*_* = 1.1E-07), rs42436495 (6:62,988,117, *P_*B*__*I*_* = 0.00005), rs42436482 (6:63,010,380, *P_*B*__*I*_* = 1.7E-07), and rs42482917 (6:64,928,624, *P_*B*__*I*_* = 2.3E-12). The changing effects of fat content of these loci were significant only when examining the interaction between SNP and lactation stage in LA1.

For the most significant region on chromosome 29, the haplotype block (29:50,229,562-50,326,170) harboring the two SNPs rs109840529 and rs110740589 contained one gene of unknown function (ENSBTAG00000050398) (Supplementary Table 8). The SNP on chromosome 1 was located in a haplotype block (1:128,226,756-128,622,561) harboring the genes *TRIM42* (tripartite motif containing 42) and *CLSTN2* (calsyntenin 2). The haplotype block around the SNP on chromosome 18 (18:53,340,459-53,596,284) included 14 genes, among them *FOXA4* (forkhead box A3) and *IGFL1 (IGF* like family member 1).

### Effects on Milk Protein Yield and Content

Highly significant effects (P_*BF*_ < 0.01) on milk protein yield were identified on chromosomes 1 and 20 with data from LA3 and LA2, respectively (Table 1 and Supplementary Figure 6). The SNPs rs43246393 (1:79,757,250, *P_*BF*_* = 0.00007, Supplementary Figure 3X) and rs110353352 (20:71,448,297, *P_*BF*_* = 0.00503, Supplementary Figure 3Y) on these two chromosomes showed besides their effects on milk protein yield also an effect on milk and fat yield in the same lactations. While the minor allele of the chromosome 1 SNP was disadvantageous for all yield traits, the minor allele of the chromosome 20 SNP was favorable.

All association tests with milk protein content resulted in highly inflated *p*-values with λ ≥ 1.5 (Supplementary Figure 2). Therefore, we decided to focus on most significant results by lowering the genome-wide significance threshold 100-fold from P_*BF*_ < 0.05 to P_*BF*_ < 0.0005. This resulted in 16 associated SNPs for protein content in different lactation periods and numbers (Supplementary Table 4 and Supplementary Figure 7). These associations were found on chromosomes 2, 3, 5, 6, 10, 18, 20, and 28. The most significant association was found on chromosome 3 (rs110474631, 3:21,764,453, *P_*BF*_* = 4.5E-06, Supplementary Figure 3Z). This SNP was found for protein content in LA1 (200, 305 days), LA2, and LAm. The minor allele G of this SNP had a frequency of 0.20 and decreased protein content by on average 0.05 % points in the before mentioned lactation periods and numbers. The corresponding haplotype block (3:21,764,453-21,819,709) does not contain any gene, but the 1 Mb region harbors 23 different genes (Supplementary Table 9). The SNP rs110291935 located on chromosome 6 was the second highest association found in all periods of LA1 (6:80,530,130, P_*BF*_ < 0.0003, Supplementary Figure 3A2). The minor allele T of this SNP increased protein content by 0.045-0.047 % points. The same SNP was already found to be associated with milk yield in all periods of LA1 (Table 1), where the minor allele decreased milk yield by 71, 132, and 209 kg in the 100, 200, and 305 days performance data, respectively. The corresponding haplotype and candidate genes were described in detail in the section of associations with milk yield. The third highest association was found on chromosome 10 at 46.5 Mb with the top SNP rs109605174 (10:46,450,562, *P_*BF*_* = 6.5E-06, Supplementary Figure 3B2) in LA2. The minor allele of this SNP increased protein content by 0.055 % points. The neighboring SNPs rs109277788 (10:44,773,979, P_*BF*_ = 0.00002) and rs43625129 (10:47,670,717, *P_*BF*_* = 9.2E-06) increased also protein content in LA2 and in the first 100 days in LA1. The corresponding haplotype block (10:46,330,098-46,672,954) comprises seven genes ENSBTAG00000054388, ENSBTAG00000050908, ENSBTAG00000019474, *FBXL22* (F-box and leucine rich repeat protein 22), *USP3* (ubiquitin specific peptidase 3), *CA12* (carbonic anhydrase 12), and *APH1B* (aph-1 homolog B, gamma-secretase subunit).

### Missing Associations to Regions With Known Effects on Milk Production

Since some genes are well known for their effects on milk yield and composition in Holstein cattle, we separately tested the association of SNPs in close proximity (<500 kb) to such candidate genes (Ogorevc et al., 2009). We found 19 SNPs on the SNP chip that were in close proximity to 10 candidate genes for milk production (*ABCG2*, *CSN1S1*, *CSN1S2*, *CSN2*, *DGAT1*, *GHR*, *GPAT4*, *PAEP*, *PRLR*, and *SPP1)*. None of those 19 SNPs were significantly or suggestively associated with any of the investigated traits in DSN (Supplementary Table 10 and Supplementary Figure 8).

### Overlap With Other Publications

Overall 31 out of 76 identified SNPs (including 16 highly significant SNPs associated with protein content) that were found to be associated with milk traits in DSN were reported in other studies (Supplementary Table 11) whose results were available in the Cattle QTLdb. Seven SNPs were found to be associated with the same milk traits in Holstein cattle as in DSN. These SNPs were located on chromosome 3 at 15 Mb (rs110073735, 3:15,470,670) and 22 Mb (rs41587408, 3:21,692,628), chromosome 6 between 86−87 Mb (rs109592101, 6:86,112,142; rs41591365, 6:87,266,808), chromosome 10 at 46 Mb (rs109605174, 10:46,450,562), chromosome 20 at 51 Mb (rs41948928, 20:50,879,180), and chromosome X at 13 Mb (rs109188619, X:12,896,716) (Kolbehdari et al., 2009; Cole et al., 2011; Meredith et al., 2012; Nayeri et al., 2016; Wang and Chatterjee, 2017; Jiang et al., 2019). Eight additional SNPs that were significant in DSN were found to be associated with other milk traits in Holstein and Brown Swiss cattle. Those were located on chromosome 5 at 75 Mb (rs110803736, 5:74,853,402), chromosome 6 between 65–81 Mb (rs42482917, 6:64,928,624; rs42224984, 6:65,962,733; rs41652041, 6:77,688,509; rs110291935, 6:80,530,130; rs109872424, 6:80,626,467), chromosome 8 at 54 Mb (rs41793393, 8:53,663,120), and chromosome 16 at 40 Mb (rs41804404, 16:40,391,486) (Poulsen et al., 2015; Buitenhuis et al., 2016; Dadousis et al., 2017; Jiang et al., 2019). Further, 16 associations to exterior, health, meat and carcass, production, reproduction traits in diverse breeds were reported (Snelling et al., 2010; Bolormaa et al., 2011; Cole et al., 2011; Hawken et al., 2012; McClure et al., 2012; Doran et al., 2014; Aliloo et al., 2015; Mészáros et al., 2015; Mapholi et al., 2016; Parker Gaddis et al., 2016; Li et al., 2017; Mateescu et al., 2017; Nayeri et al., 2019). When expanding the region of interest not only to single SNPs reported in this study, but to 1 Mb regions centered at the respective SNPs, all of the regions identified in DSN overlap with associations uploaded to the Cattle QTLdb.

Especially, for the region on chromosome 6 between 80.5–87.2 Mb (flanking SNPs: rs110291935 and rs41591365), which was significantly associated with milk and protein yield in all investigated periods of LA1 in our study, many different associations could be found in Cattle QTLdb. These associations included milk and protein yield as well as milk kappa-casein content in Holstein cattle (Meredith et al., 2012; Buitenhuis et al., 2016; Jiang et al., 2019), curd firmness and cheese fat recovery in Brown Swiss cattle (Dadousis et al., 2017), health traits such as somatic cell score in Holstein cattle (Jiang et al., 2019), and exterior traits such as facial pigmentation in Fleckvieh cattle (Mészáros et al., 2015).

## Discussion

In this study, we investigated genetic factors underlying milk production traits in DSN cattle. We detected 41 significant and 20 suggestive SNPs mostly found in regions previously associated to milk production traits or other trait categories. The high overlap of associated SNPs in this study with other studies shows that the regions that influence milk traits in DSN may have similar or even pleiotropic functions in other breeds.

We observed a high overlap of identified genomic regions in DSN and Holstein, even if we did not find significant effects of major candidate genes that affect milk production in Holstein cattle. While the key genes driving e.g., milk yield or fat and protein content might be different between DSN and Holstein, it is surprising that especially the well described association in the region of and around *DGAT1* was not detected in DSN although DSN and Holstein cattle are closely related. Although the MAFs of the three closest SNPs to *DGAT1* gene are high (MAF = 0.44–0.47) in DSN (Supplementary Table 10), we observed that the actual variant causing the A232K substitution is very rare having a MAF of 0.02 (preliminary results of sequencing data from 57 DSN cattle). It is possible that not only the causal variant of *DGAT1*, but also of other known milk genes are rare in DSN and thus do not have much effect in the DSN population.

Several interesting candidate genes were found including *CSN3* gene (kappa casein) that belongs to the casein cluster on chromosome 6 at 86 Mb, which are known to influence milk fat and protein content as well as milk properties (Caroli et al., 2009), also plays an important role in DSN for milk and protein yield. The genetic architecture of the casein gene cluster of DSN in comparison to other breeds was recently investigated in detail (Meier et al., 2019). Among 14 investigated breeds in that study, the casein gene cluster of DSN was most similar to Danish Red. One of the most significant associations on chromosome 1 at 79 Mb was close to *SST* (somatostatin) and *BCL6* (BCL6 transcription repressor). Since *SST* regulates the secretion of pituitary hormones including prolactin (Eigler and Ben-Shlomo, 2014), it may contribute to the regulation of milk production (Dybus, 2002; Alipanah et al., 2007). And *BCL6* was found to be expressed in mammary epithelium and also in breast cancer (Logarajah et al., 2003). Also interesting is *SLC6A3* gene (solute carrier family 6 member 3) on chromosome 20 at 71 Mb to which the Gene Ontology term “lactation” (GO:0007595) was assigned. This assignment was inferred from electronic annotation by Gene Ontology Consortium (Ashburner et al., 2000) and is possibly based on the homology of this gene in mice where it was shown that mutations in this gene cause lactation failure (Bossé et al., 1997). Beside *SLC6A3*, other solute carrier family genes were found in the same region: *SLC6A18* (family 6 member 18), *SLC6A19* (family 6 member 19), SLC9A3 (family 9 member 3), and *SLC12A7* (family 12 member 7). Also, genes coding for enzymes or receptors targeting milk ingredients such as prostaglandin and riboflavin were found, e.g., *PTGR1* (prostaglandin reductase 1) on chromosome 8 at 101 Mb, *PTGIR* (prostaglandin I2 receptor) on chromosome 18 at 54 Mb, and *FLAD1* (flavin adenine dinucleotide synthetase 1) on chromosome 3 at 15 Mb.

Since population stratification has a significant effect on the number of false positive QTLs detected during association analysis, we tried multiple approaches to reduce the inflation factor λ for different traits of interest. The higher the inflation factor λ, the higher the number of false-positively reported significant associations in GWAS analyses. Although we tried to compensate for population stratification by using pairwise population concordance test and paternal pedigree information, we were unable to capture the population structure entirely. That is most likely due to the fact that the population size of DSN is small, and the number of breeding bulls used each generation is limited. As such, DSN animals are not unrelated and complex family relationships exist between animals, which is a common issue in small populations. Thus, we assume that the residual inflation is genetically caused due to high linkage disequilibrium between SNPs. Moreover, using both population stratification and pedigree information led to an overfitting of the model that took away genetic variance. Further genetic variance was lost as we required that each sire was represented by at least 20 offspring in the phenotype data and thus the number of sires contributing to the analyzed DSN population was reduced by two-thirds. By doing so, also the number of animals available for GWAS was reduced and as a result the statistical power to detect significant associations. Nonetheless, we believe that lowering the genetic variance and statistical power in favor of lower inflation of *p*-values is the proper way to prevent false positive associations.

The results of this study could be used as prior information to detect SNPs associated with milk production traits in other (related) breeds. For that, cattle from other breeds could be genotyped for single SNPs (e.g., top SNPs) instead of being genotyped using SNP chips or even being whole genome sequenced which would reduce costs. Furthermore, since genotypic data of a sufficient amount of DSN cattle was not available yet, genomic breeding is not yet established. The identified SNPs and their associations could be directly used to make breeding decisions by DSN breeders. Even predictions about performance of young DSN cows that do not have any phenotypic data yet could be made if genotypic data would be available. Thus, a first step toward genomic breeding for milk traits in DSN cattle was made. Further analyses are needed to evaluate whether these associated SNPs do not show any negative effect on other trait categories such as fertility.

## Conclusion

The genome-wide association analysis identified several significant regions associated with milk traits in diverse lactation periods and numbers in dual-purpose DSN cattle. In the view of the fact that the biggest possible sample size of the investigated DSN population was still relatively small, the most promising SNPs for improving milk production in DSN are those that were also significantly associated with milk traits in other studies. These SNPs were located on chromosome 3, 5, 6, 8, 10, 20, and X. In contrast, no association to the well-known *DGAT1* region could be detected in DSN. Further analysis is needed to make sure that these SNPs do not negatively affect other important production traits or DSN characteristic traits such as carcass and meat, conformation, fertility, and health before using them as breeding markers. Nevertheless, the results of this study are a basis for further genetic analysis to identify genes and causal variants that affect milk traits in DSN cattle as well as in other related breeds.

## Data Availability Statement

The datasets presented in this study can be found in online repositories. The names of the repositories and accession numbers can be found below: The European Molecular Biology Laboratory’s European Bioinformatics Institute (EMBL-EBI) European Nucleotide Archive (ENA) and European Variation Archive (EVA), https://www.ebi.ac.uk/ena/browser/home and https://www.ebi.ac.uk/eva/, PRJEB42513 (project) and ERZ1701738 (analyses).

## Ethics Statement

Ethical review and approval was not required for the animal study because samples were collected based on routine procedures on these farm animals. Ear tags were taken as part of the required registration procedure, blood samples were taken by a trained veterinarian to perform standard health recording. Written informed consent was obtained from the owners for the participation of their animals in this study.

## Author Contributions

GB, PK, and DA designed the study. PK performed all computational and statistical analysis, interpreted the data, and drafted the manuscript. DA helped with the statistical analysis. SK and KM provided genotypes of 61 cows. DA and GB helped draft the manuscript. All authors read and approved the final manuscript.

## Conflict of Interest

The authors declare that the research was conducted in the absence of any commercial or financial relationships that could be construed as a potential conflict of interest.
