# Supplementary material for: Genomic Loci Affecting Milk Production in German Black Pied Cattle (DSN)
Source: Front Genet. 2021 Mar 8;12:640039. doi: 10.3389/fgene.2021.640039 (PMC7982544; doi:10.3389/fgene.2021.640039)
Supplement: Supplementary file 1 [file Data_Sheet_1.PDF]

Supplementary Material

Genomic loci affecting milk production in German Black Pied cattle (DSN)

Paula Korkuć[1], Danny Arends[1], Katharina May[2], Sven König[2], Gudrun A. Brockmann[1]*

[1] Humboldt University Berlin, Albrecht Daniel Thaer- Institute for Agricultural and Horticultural Sciences, Animal Breeding Biology, and Molecular Genetics, Invalidenstr. 42, 10115 Berlin, Germany
[2] Justus-Liebig-University of Gießen, Institute of Animal Breeding and Genetics, Ludwigstr. 21, 35390 Gießen, Germany

*Correspondence:
Gudrun Brockmann
gudrun.brockmann@hu-berlin.de

Content

1.1 GWAS model and covariates 2
1.2 Q-Q-plots 4
1.3 GWAS results for protein content (λ≥1.5) 4
1.4 Correlation of traits 5
1.5 SNP effect plots 6
1.6 Interaction between lactation and SNP genotype 8
1.7 Haplotypes and genes 10
1.8 Manhattan plots 17
1.9 Comparison to significant SNPs for milk production traits in Holstein 21
1.10 Comparison to publications 22

1.1 GWAS model and covariates

Supplementary Table 1: Overview of tested GWAS models and the corresponding inflation factor λ. Inflation factor λ is shown for milk kg and protein % in LA1 (305d), and as average ($\bar{\lambda}$) over all traits in LA1-LA3 (305d). The model m_0 included the covariates for population stratification ps_i, farm f_j, sire s_k, birth year by_l, birth season bs_m, calving year cy_n, calving season cs_o, age at first calving in days ac_p, and the SNP genotype g_q as fixed effects for each investigated trait y. Covariates marked with an asterisk "*" were only included into the model, if the difference in the Akaike information criterion ΔAIC was ≤ -10 between the null model ($y_i = ps_i$) and the null model extended with one of the covariates ($y_{ix} = ps_i + covarite_x$). The lowest inflation factor λ was observed for model m_5, which was selected for GWAS in this study (highlighted in green).

**Model number**	**Model adjustment**	**Milk kg (LA1)**	**Protein % (LA1)**	**All phenotypes (LA1-LA3)**
		**λ or $\bar{\lambda}$ (difference to m₀)**		
m_0	$y_{ijlmnopq} = ps_i + f_j^* + by_l^* + bs_m^* + cy_n^* + cs_o^* + ac_p^* + g_q + error_{ijlmnopq}$	1.97	2.85	1.80
m_1	m_0 only with SNP genotype ($y_q = g_q + error_q$)	5.65 (+3.68)	5.57 (+2.72)	4.62 (+2.82)
m_2	m_0 with substructure based on EMMA relationship matrix (Hyun et al., 2008)	2.08 (+0.11)	2.93 (+0.08)	1.92 (+0.12)
m_3	m_0 with substructure based on PC1, PC2, and PC3 (9.8%, 9.0%, 8.1% variance explained respectively)	2.05 (+0.08)	3.35 (+0.50)	2.19 (+0.39)
m_4	m_0 using biggest farm only (~850 cows in LA1)	1.73 (-0.24)	2.58 (-0.27)	1.68 (-0.12)
m_5	m_0 with additional covariate sire s_k	1.38 (-0.59)	2.01 (-0.84)	1.49 (-0.31)
m_6	traits pre-corrected with covariates, then model only with SNP genotype ($corrected\ y_q = g_q + error_q$)	1.95 (-0.02)	2.82(-0.03)	1.80 (+0.00)
m_7	m_0, traits not filtered for min. 20 animals per farm, per sire, and per birth year	2.30 (+0.33)	2.99 (+0.14)	1.96 (+0.16)
m_8	m_0 with GEMMA software and additional covariate sire s_k	1.52 (-0.45)	2.18 (-0.67)	1.56 (-0.24)

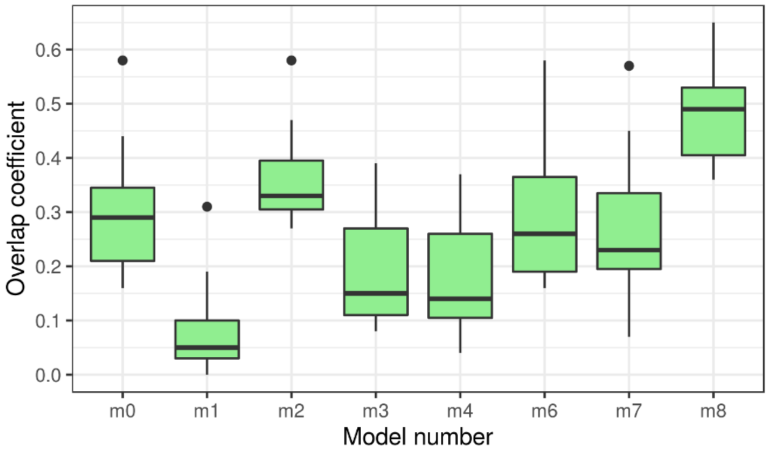

Supplementary Figure 1: Comparison of top 100 SNPs between selected model m_5 and residual models listed in Supplementary Table 1 using overlap coefficient. The overlap coefficient is defined as size of the intersection divided by the size of the smaller of the two sets. Only SNPs between models investigating the same trait were compared to each other. The average overlap coefficient was 0.27 (±0.15 sd) meaning that on average 27% of top 100 SNPs are shared between the pairwise tested GWAS models. The highest average overlap coefficient of 0.48 was calculated between the top 100 SNPs of model m_5 and m_8, since they both included sire as covariate.

Supplementary Table 2: Difference in Akaike information criterion (ΔAIC) between null model ($\mathrm{y_i = ps_i}$) and the null model extended with one of the covariates ($\mathrm{y_{ix} = ps_i + covarite_x}$) for each investigated trait y. If ΔAIC was ≤ -10, the covariate was included into the model (highlighted in bold). Covariates calving year and calving season could not be applied to traits for lactation mean (LAm) and thus are marked as not available ("NA").

Trait	Farm	Sire	Birth year	Birth season	Calving year	Calving season	Age at first calving (days)
**Milk kg (100d)**	**-387.8**	**-76.8**	**-24.5**	**-17.8**	**-17.0**	**-28.1**	**-63.3**
**Milk kg (200d)**	**-441.4**	**-67.0**	**-20.5**	**-28.4**	**-13.3**	**-28.6**	**-81.5**
**Milk kg (LA1)**	**-353.9**	**-51.5**	**-12.1**	**-20.7**	-5.1	**-11.5**	**-73.6**
**Milk kg (LA2)**	**-432.0**	**-16.1**	9.8	**-17.4**	4.9	**-28.0**	**-101.4**
**Milk kg (LA3)**	**-379.4**	-1.2	11.4	-6.0	10.1	**-27.5**	**-134.1**
**Milk kg (LAm)**	**-286.9**	10.3	4.8	**-13.6**	NA	NA	**-75.1**
**Fat kg (100d)**	**-366.3**	**-57.3**	**-24.0**	-5.0	**-24.3**	-7.3	**-50.1**
**Fat kg (200d)**	**-490.7**	**-55.9**	**-27.6**	-3.7	**-12.9**	**-50.6**	**-81.8**
**Fat kg (LA1)**	**-453.2**	**-44.1**	**-17.5**	-5.9	**-15.3**	-2.9	**-81.7**
**Fat kg (LA2)**	**-482.1**	**-26.9**	-6.5	**-14.4**	**-10.1**	**-22.2**	**-100.5**
**Fat kg (LA3)**	**-393.3**	-1.0	3.8	-1.8	5.4	**-24.3**	**-142.8**
**Fat kg (LAm)**	**-346.8**	7.5	3.2	-9.0	NA	NA	**-92.2**
**Protein kg (100d)**	**-582.0**	**-86.3**	**-34.0**	**-19.6**	**-25.6**	**-13.9**	**-122.9**
**Protein kg (200d)**	**-670.3**	**-75.4**	**-30.6**	**-11.3**	**-22.3**	-7.3	**-148.3**
**Protein kg (LA1)**	**-513.7**	**-40.9**	**-13.3**	-9.6	**-6.2**	-1.9	**-110.9**
**Protein kg (LA2)**	**-579.1**	**-15.1**	11.2	-9.9	1.9	**-21.4**	**-127.8**
**Protein kg (LA3)**	**-487.4**	-3.1	11.4	-4.0	10.3	**-20.1**	**-160.2**
**Protein kg (LAm)**	**-412.4**	8.0	5.8	-8.4	NA	NA	**-104.4**
**Fat % (100d)**	**-43.8**	-6.1	**-15.2**	**-15.6**	-3.1	**-65.2**	-0.8
**Fat % (200d)**	**-85.8**	**-41.7**	**-22.4**	**-25.5**	**-12.9**	**-50.6**	-1.6
**Fat % (LA1)**	**-108.7**	**-72.9**	**-21.4**	-7.0	-7.2	-6.4	1.8
**Fat % (LA2)**	**-120.3**	**-23.4**	-4.4	5.7	-6.8	4.0	1.3
**Fat % (LA3)**	**-60.5**	**-10.1**	-9.4	4.2	-3.6	5.3	1.1
**Fat % (LAm)**	**-63.5**	11.6	1.5	5.8	NA	NA	1.9
**Protein % (100d)**	**-168.2**	-8.5	4.1	**-61.8**	-6.3	**-202.6**	**-44.9**
**Protein % (200d)**	**-218.1**	**-19.4**	0.2	**-50.1**	-9.4	**-130.0**	**-52.2**
**Protein % (LA1)**	**-201.4**	**-45.7**	0.5	**-14.9**	-2.7	**-27.3**	**-37.5**
**Protein % (LA2)**	**-123.8**	-3.6	13.4	-6.4	-1.3	**-21.9**	-7.2
**Protein % (LA3)**	**-111.6**	-4.8	7.9	-0.4	-6.2	-0.5	-4.9
**Protein % (LAm)**	**-104.4**	14.8	12.2	-0.4	NA	NA	**-13.7**

Supplementary Table 3: Difference in Akaike information criterion (ΔAIC) between null model ($\mathrm{y_s} = (1|\mathrm{animal}_s)$) and the null model extended with one of the covariates ($\mathrm{y_{xs} = covariate_x} + (1|animal_s)$ for each investigated trait y. If ΔAIC was ≤ -10, the covariate was included into the model (highlighted in bold).

Performance data	Trait	Farm	Sire	Birth year	Birth season	Calving year	Calving season	Age at first calving (days)
**100-305d**	Milk kg	**-640.4**	**-627.0**	**-87.6**	**-48.1**	**-77.5**	**-45.4**	**-4222.3**
	Fat kg	**-687.9**	**-382.6**	**-35.0**	**-15.2**	**-27.5**	**-18.0**	**-2061.3**
	Protein kg	**-838.8**	**-448.3**	**-30.8**	**-16.6**	**-17.5**	**-14.0**	**-2084.6**
	Fat %	**-114.4**	**-117.4**	17.8	14.8	21.7	-3.3	724.3
	Protein %	**-248.6**	-1.6	54.9	-7.1	39.2	**-66.8**	1032.0
**LA1-3**	Milk kg	**-812.6**	**-692.6**	**-101.1**	**-50.2**	**-686.9**	**-79.1**	**-5140.6**
	Fat kg	**-844.2**	**-461.5**	**-56.1**	**-23.8**	**-547.7**	**-45.2**	**-2971.0**
	Protein kg	**-992.3**	**-499.7**	**-41.1**	**-24.4**	**-656.3**	**-46.0**	**-2955.1**
	Fat %	**-193.0**	**-199.1**	41.7	19.1	**-63.1**	-1.0	734.2
	Protein %	**-239.5**	**-39.3**	62.3	15.1	**-143.5**	**-40.2**	1192.6

1.2 Q-Q-plots

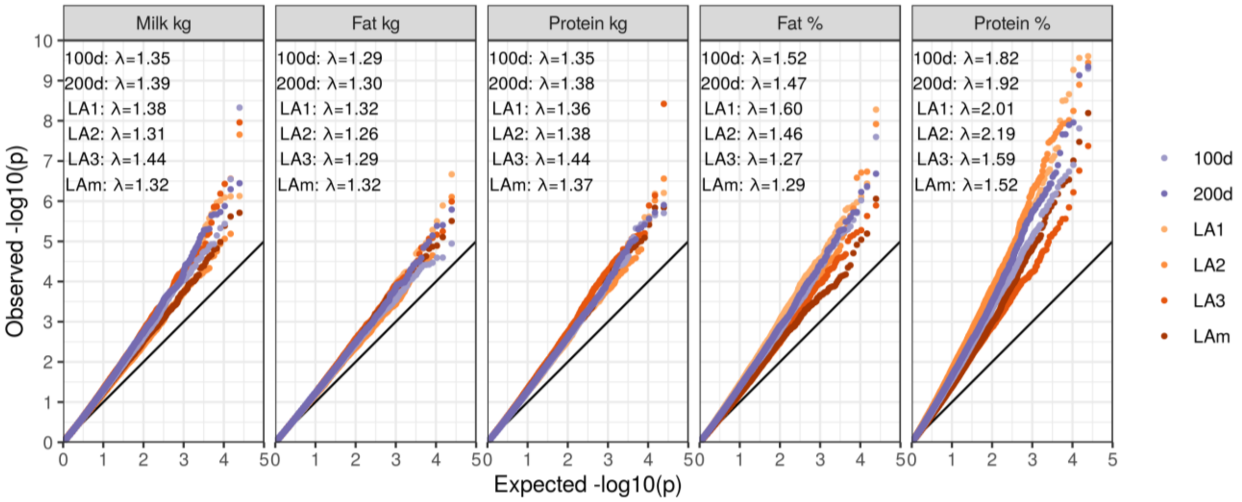

Supplementary Figure 2: Q-Q-plots for milk performance traits in DSN. Plots show the expected -$\log_{10}$(p-values) on the x-axis and the observed -$\log_{10}$(p-values) on the y-axis for milk, fat, and protein yield in kg, and fat and protein content in percentage (left to right) of 100d and 200d performance data of LA1 (light to dark violet) and 305d data of LA1-LA3 and the lactation mean (LAm) (light to dark orange). Inflation factor λ is provided for each investigated trait in the individual plots.

1.3 GWAS results for protein content (λ≥1.5)

Supplementary Table 4: GWAS for milk production traits in DSN which had an inflation factor λ≥1.5 (see Supplementary Figure 2). For each trait, significantly associated SNPs are listed with chromosome number (Chr), chromosomal position in base pairs, the SNP reference ID (SNP-ID), the given allele on the forward strand in the reference genome ARS-UCD1.2 (Ref), the alternative allele (Alt), the minor allele in the examined DSN population (MA), and the minor allele frequency (MAF). Furthermore, the associated trait, the number of cows in the specific analysis of this trait (n), the allele substitution effect of the minor allele (regression coefficient β), the standard error (SE) of β, and the p-value after Bonferroni correction (P_{BF}) are given. P_{BF}<0.0005 was considered significant. SNPs that were significant for traits in Table 1 are highlighted in bold.

Chr	Position (bp)	SNP-ID	Ref	Alt	MA	MAF	Trait	n	β	SE(β)	P_{BF}
2	128,295,010	rs41629000	C	T	T	0.40	Protein % (LA2)	1149	0.046	0.008	0.00033
3	15,470,670	rs110073735	C	T	T	0.17	Protein % (LA2)	1124	-0.066	0.011	0.00018
	21,692,628	rs41587408	T	C	T	0.19	Protein % (LA1)	1364	-0.053	0.014	9.9E-06
							Protein % (200d LA1)	1471	-0.055	0.015	0.00020
	21,764,453	rs110474631	G	A	G	0.20	Protein % (LA1)	1368	-0.05	0.014	4.5E-06
							Protein % (LA2)	1151	-0.05	0.016	0.00037
							Protein % (LAm)	680	-0.048	0.020	0.00012
							Protein % (200d LA1)	1476	-0.053	0.015	0.00023
	26,278,860	rs109138764	A	G	G	0.10	Protein % (LA1)	1357	0.076	0.013	0.00018
5	74,853,402	rs110803736	C	T	T	0.41	Protein % (LA1)	1357	-0.045	0.008	0.00006
							Protein % (200d LA1)	1463	-0.048	0.008	0.00001
6	65,962,733	rs42224984	C	T	T	0.30	Protein % (LA2)	1108	0.049	0.010	0.00027
	**80,530,130**	**rs110291935**	**T**	**C**	**T**	**0.41**	Protein % (LA1)	1366	0.047	0.008	5.0E-06
							Protein % (100d LA1)	1475	0.045	0.008	0.00028
							Protein % (200d LA1)	1474	0.047	0.008	8.2E-06
10	34,947,852	rs41601192	T	C	C	0.38	Protein % (LA2)	1146	0.046	0.009	0.00021
							Protein % (200d LA1)	1469	0.046	0.008	0.00040
	35,164,676	rs29016462	G	A	A	0.29	Protein % (LA2)	1147	0.051	0.010	0.00022

	44,773,979	rs109277788	C	T	T	0.32	Protein % (LA2)	1151	0.057	0.010	0.00002
	46,450,562	rs109605174	C	G	C	0.35	Protein % (LA2)	1145	0.055	0.009	6.5E-06
	47,670,717	rs43625129	G	A	A	0.42	Protein % (100d LA1)	1450	0.052	0.008	9.2E-06
18	33,540,904	rs41869985	G	T	G	0.43	Protein % (LA2)	1142	-0.045	0.008	0.00047
20	50,879,180	rs41948928	T	C	T	0.10	Protein % (LA1)	1356	-0.081	0.013	0.00005
							Protein % (LA2)	1145	-0.084	0.014	0.00010
							Protein % (200d LA1)	1464	-0.078	0.014	0.00040
28	18,458,637	rs110007227	T	C	C	0.26	Protein % (LA1)	1364	-0.047	0.011	0.00006

1.4 Correlation of traits

Supplementary Table 5: Squared correlation between traits milk, fat, and protein yield. Correlation coefficients r^2 were calculated for traits of the 305-days (305d) performance of lactation 1 (LA1), lactation 2 (LA2), lactation 3 (LA3), the lactation mean (LAm), and the 100 days (100d) and 200 days (200d) performance of LA1. Correlation coefficients ≥ 0.8 are highlighted in bold red.

		Milk kg						Fat kg						Protein kg					
		100d LA1	200d LA1	LA1	LA2	LA3	LAm	100d LA1	200d LA1	LA1	LA2	LA3	LAm	100d LA1	200d LA1	LA1	LA2	LA3	LAm
Milk kg	100d LA1																		
	200d LA1	0.93																	
	LA1	0.82	0.93																
	LA2	0.45	0.53	0.57															
	LA3	0.42	0.50	0.53	0.65														
	LAm	0.61	0.71	0.77	0.87	0.87													
Fat kg	100d LA1	0.73	0.68	0.60	0.36	0.34	0.49												
	200d LA1	0.72	0.76	0.71	0.44	0.43	0.60	0.90											
	LA1	0.65	0.72	0.78	0.48	0.47	0.65	0.76	0.92										
	LA2	0.33	0.38	0.41	0.79	0.54	0.68	0.39	0.50	0.56									
	LA3	0.31	0.36	0.39	0.53	0.82	0.69	0.34	0.44	0.49	0.65								
	LAm	0.46	0.52	0.58	0.72	0.73	0.81	0.53	0.68	0.75	0.88	0.86							
Protein kg	100d LA1	0.86	0.83	0.74	0.47	0.47	0.63	0.75	0.77	0.69	0.41	0.40	0.55						
	200d LA1	0.81	0.89	0.84	0.54	0.55	0.72	0.70	0.82	0.78	0.47	0.46	0.63	0.93					
	LA1	0.72	0.83	0.91	0.55	0.55	0.75	0.61	0.76	0.85	0.49	0.47	0.67	0.81	0.92				
	LA2	0.41	0.48	0.52	0.92	0.65	0.82	0.38	0.48	0.53	0.84	0.61	0.79	0.50	0.59	0.61			
	LA3	0.37	0.44	0.47	0.60	0.93	0.79	0.33	0.44	0.48	0.57	0.86	0.77	0.48	0.57	0.58	0.70		
	LAm	0.53	0.62	0.68	0.79	0.82	0.91	0.49	0.62	0.68	0.72	0.76	0.87	0.66	0.76	0.80	0.89	0.89	

Supplementary Table 6: Squared correlation between traits fat and protein content. Correlation coefficients r^2 were calculated for traits of the 305-days (305) performance data of lactation 1 (LA1), lactation 2 (LA2), lactation 3 (LA3), the lactation mean (LAm), and the 100 days (100d) and 200 days (200d) performance of LA1. Correlation coefficients ≥ 0.8 are highlighted in bold red.

		Fat %					
		100d LA1	200d LA1	LA1	LA2	LA3	LAm
Fat %	100d LA1						
	200d LA1	0.73					
	LA1	0.55	0.90				
	LA2	0.24	0.43	0.51			
	LA3	0.19	0.35	0.43	0.53		
	LAm	0.32	0.63	0.77	0.83	0.78	

		Protein %					
		100d LA1	200d LA1	LA1	LA2	LA3	LAm
Protein %	100d LA1						
	200d LA1	0.81					
	LA1	0.66	0.91				
	LA2	0.33	0.48	0.57			
	LA3	0.27	0.45	0.54	0.60		
	LAm	0.47	0.71	0.83	0.85	0.83	

1.5 SNP effect plots

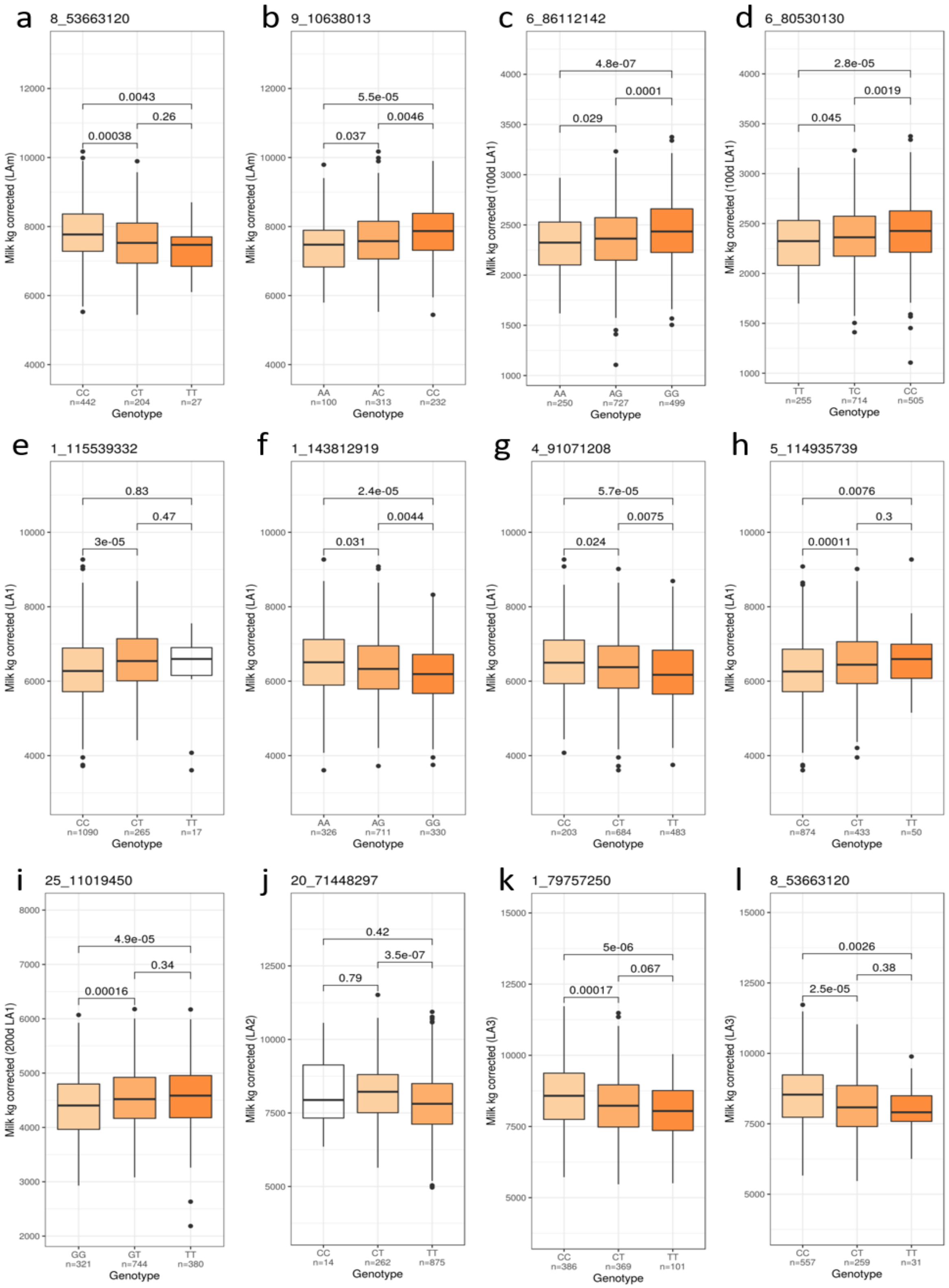

Supplementary Figure 3: SNP effect plots of associated SNPs mentioned in the manuscript. They show the corrected phenotypes for SNP genotype groups (homozygous reference allele, heterozygous, and homozygous to the alternative allele) and the number of animals (n) falling into the individual genotype groups. P-values between genotype groups from two-sided t-tests are displayed. Plots are ordered chronologically with regard to their first mention in the manuscript. Genotype groups with less than 30 observations were not used in order to prevent spurious associations, thus they are only shown for the sake of completeness (white boxplots).

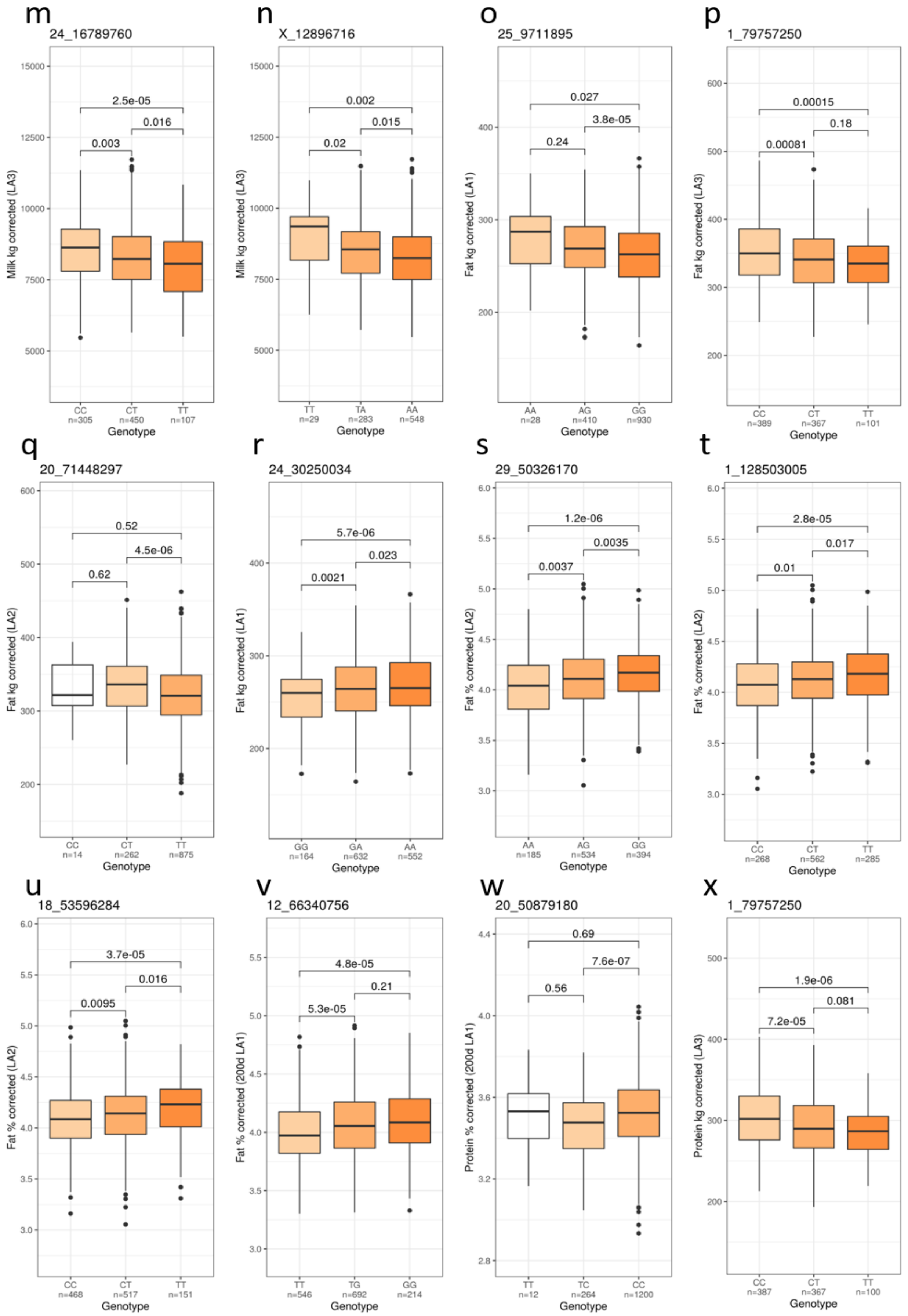

Continuation of Supplementary Figure 3: SNP effect plots of associated SNPs mentioned in the manuscript. Plots are ordered chronologically with regard to their first mention in the manuscript.

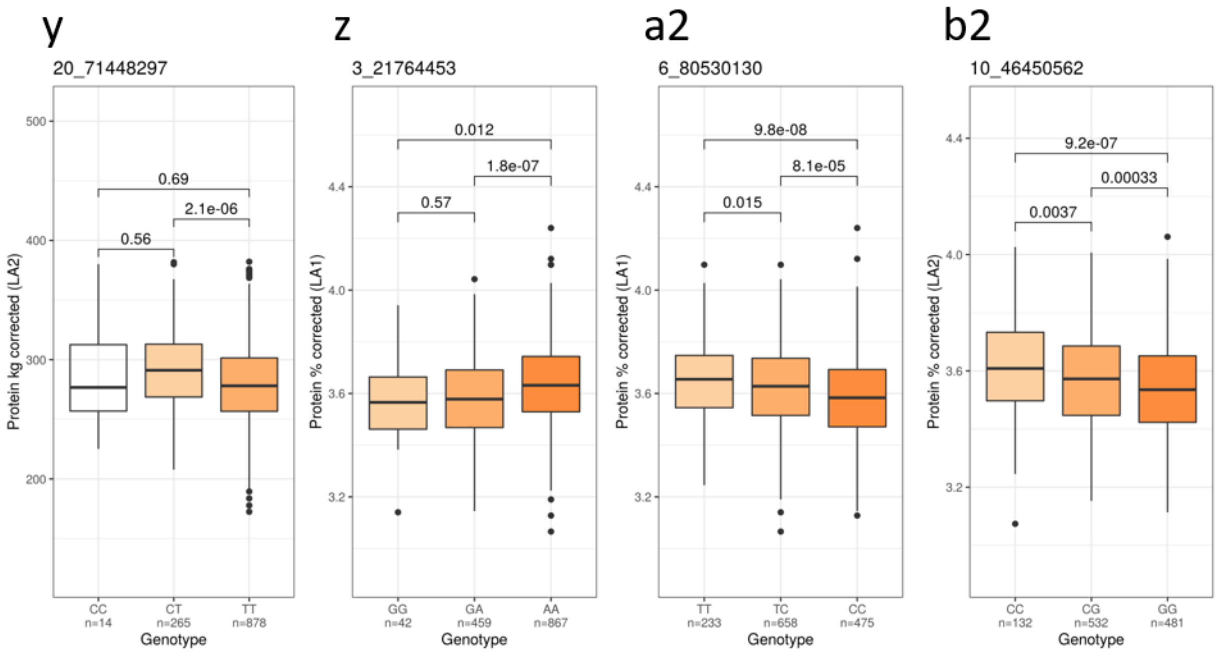

Continuation of Supplementary Figure 3: SNP effect plots s of associated SNPs mentioned in the manuscript. Plots are ordered chronologically with regard to their first mention in the manuscript.

1.6 Interaction between lactation and SNP genotype

Supplementary Table 7: GWAS results of the interaction term lactation x SNP genotype. Interaction analysis was performed for all lactation stages in LA1 (stage) and all lactation numbers (LA) of 305d performance data for milk, fat, and protein yield, and fat and protein content. Only SNPs were tested which were listed in Table 1. SNPs are listed with the SNP-ID, the traits from Table 1 they were significant for, the investigated term, and the Bonferroni-corrected p-values (P_{BF}) of the SNP and the interaction term. SNP results that are carried over from Table 1 are highlighted in grey.

SNP (chr:pos)	SNP-ID	Trait	Investigated term	P_{BF} of SNP	P_{BF} of interaction
1:79,757,250	rs43246393	Milk kg (LA3)	SNP	0.00020	NA
		Fat kg (LA3)	SNP	0.01868	NA
		Protein kg (LA3)	SNP	0.00007	NA
		Protein kg (LAm)	SNP	0.02641	NA
		Milk kg (100-305d)	SNP + SNP x stage	1.00000	0.00001
		Fat kg (100-305d)	SNP + SNP x stage	1.00000	0.00008
		Protein kg (100-305d)	SNP + SNP x stage	1.00000	0.00329
		Milk kg (LA1-3)	SNP + SNP x LA	1.00000	0.00023
		Fat kg (LA1-3)	SNP + SNP x LA	1.00000	0.00063
		Protein kg (LA1-3)	SNP + SNP x LA	1.00000	0.02849
1:128,503,005	rs109686415	Fat % (LA2)	SNP	0.00357	NA
		Fat % (LAm)	SNP	0.01599	NA
		Fat % (100-305d)	SNP + SNP x stage	1.00000	0.00016
4:47,674,986	rs110143001	Fat % (LA3)	SNP	0.04275	NA
		Protein % (100-305d)	SNP + SNP x stage	1.00000	0.00626
5:83,959,138	rs41660560	Protein kg(LA2)	SNP	0.01767	NA
		Fat % (100-305d)	SNP + SNP x stage	1.00000	0.00003
5:93,953,629	rs109945272	Fat %(LA2)	SNP	0.08780	NA
		Fat % (100-305d)	SNP + SNP x stage	1.00000	1.9E-06
6:60,162,206	rs41605188	Milk kg (LA1, 200d)	SNP	0.09377	NA
		Fat % (100-305d)	SNP + SNP x stage	1.00000	1.1E-07
6:62,988,117	rs42436495	Milk kg (LA1, 200d)	SNP	0.03921	NA

		Milk kg (LA1)	SNP	0.01789	NA
		Fat % (100-305d)	SNP + SNP x stage	1.00000	0.00005
6:63,010,380	rs42436482	Milk kg (LA1, 200d)	SNP	0.09298	NA
		Milk kg (LA1)	SNP	0.04884	NA
		Fat % (100-305d)	SNP + SNP x stage	1.00000	1.7E-07
6:64,928,624	rs42482917	Milk kg (LA1, 200d)	SNP	0.09593	NA
		Fat % (100-305d)	SNP + SNP x stage	1.00000	2.3E-12
		Protein % (100-305d)	SNP + SNP x stage	0.55856	0.00051
16:30,668,830	rs43041491	Fat %(LA3)	SNP	0.02338	NA
		Fat % (100-305d)	SNP + SNP x stage	1.00000	0.02478
20:50,879,180	rs41948928	Fat % (LA1, 200d)	SNP	0.00777	NA
		Protein % (100-305d)	SNP + SNP x stage	6.2E-07	0.00021
29:50,217,955	rs109241029	Fat % (LA2)	SNP	0.04229	NA
		Fat % (100-305d)	SNP + SNP x stage	1.00000	0.00345
29:50,260,533	rs109840529	Fat % (LA2)	SNP	0.00338	NA
		Fat % (100-305d)	SNP + SNP x stage	1.00000	1.4E-07

1.7 Haplotypes and genes

Supplementary Table 8: List of genes located in the haplotype block or within 1 Mb of the respective SNP in DSN from Table 1. Regions are listed as chromosome:start:stop in bp together with the SNPs, the trait which the SNPs was associated with and the genes falling into the haplotype blocks. Genes are listed with their gene name or Ensembl ID if no gene name was available. If consecutive haplotype or 1 Mb gene regions overlapped, they were merged together.

Significant SNP	Traits	Haplotype block	Genes in haplotype block	1Mb region	Additional genes in 1 Mb region
1:77,049,090	Milk kg (LA3), Protein kg (LA3)	1:76,984.280-77,049,090	*CLDN1*	1:76,549,090-77,549,090	*IL1RAP, TMEM207, CLDN16, P3H2, TP63*
1:79,757,250	Milk kg (LA3), Protein kg (LA3), Protein kg (LAm), Fat kg (LA3)	1:79,606,618-79,757,250	*BCL6, RTP2, SST*	1:79,257,250-80,257,250	*RTP4, MASP1, RTP1, ST6GAL1*
1:115,539,332	Milk kg (LA1), Protein kg (LA1)	1:115,487,178-115,649,771	*MBNL1*	1:115,039,332-116,039,332	*ENSBTAG00000033740, SUCNR1, ENSBTAG00000049884, AADAC*
1:128,503,005	Fat % (LA2), Fat % (LAm)	1:128,226,756-128,622,561	*TRIM42, CLSTN2*	1:128,003,005-129,003,005	*SLC25A36, ENSBTAG00000052041*
1:143,812,919	Milk kg (LA1), Protein kg (LA1)	1:143,783,819-143,812,919	*PDXK*	1:143,312,919-144,312,919	*CRYAA, ENSBTAG00000011437, HSF2BP, RRP1B, CSTB, RRP1, AGPAT3, TRAPPC10, ENSBTAG00000007105, GATD3A, ENSBTAG00000014880, ENSBTAG00000014882, AIRE, ENSBTAG00000051579, PFKL*
2:123,100,345	Protein kg (200d LA1)	2:123,070,905-123,172,270	-	2:122,600,345-123,600,345	*PUM1, SDC3, LAPTM5, MATN1*
3:15,947,663	Fat % (200d LA1)	3:15,947,663-16,015,122	*ADAR, ENSBTAG00000048725, CHRNB2*	3:15,447,663-16,447,663	*DPM3, SLC50A1, EFNA1, EFNA3, EFNA4, ADAM15, DCST1, ZBTB7B, FLAD1, CKS1B, SHC1, PBXIP1, PMVK, KCNN3, UBE2Q1, TDRD10, SHE, IL6R, ATP8B2, AQP10, HAX1, UBAP2L, C3H1orf43, C3H1orf189, TPM3, NUP210L, ENSBTAG00000024487*
4:47,674,986	Fat % (LA3)	4:47,266,182-47,697,476	*NAMPT*	4:47,174,986-48,174,986	*SYPL1, CCDC71L, PIK3CG, PRKAR2B*
4:91,071,208	Milk kg (LA1)	4:91,071,208-91,148,043	*GRM8*	4:90,571,208-91,571,208	-
5:62,094,191	Fat % (LA3)	5:62,094,191-62,128,735	-	5:61,594,191-62,594,191	*ENSBTAG00000045919*

5:83,959,138	Protein kg (LA2)	5:83,889,138-84,029,138	*RASSF8*	5:83,459,138-84,459,138	*ITPR2, SSPN, BHLHE41, LMNTD1*
5:93,953,629	Fat % (LA2)	5:93,840,983-93,953,629	*DERA, ENSBTAG00000023487*	5:93,453,629-94,453,629	*MGST1, SLC15A5, STRAP, EPS8, PTPRO*
5:114,935,739	Milk kg (LA1),<br>Milk kg (100d LA1),<br>Milk kg (200d LA1),<br>Protein kg (LA1),<br>Protein kg (100d LA1),<br>Protein kg (200d LA1),<br>Fat kg (200d LA1)	5:114,898,265-115,075,918	*PARVG, SHISAL1*	5:114,435,739-115,435,739	*EFCAB6, SULT4A1, ENSBTAG00000045746, ENSBTAG00000003367, SAMM50, PARVB, RTL6, PRR5, ENSBTAG00000008858, PHF21B*
6:60,162,206	Milk kg (200d LA1)	6:60,092,206-60,232,206	*APBB2, UCHL1, LIMCH1*	6:59,662,206-60,662,206	*RBM47, NSUN7, ENSBTAG00000051208, PHOX2B*
6:61,733,082	Milk kg (LA1),<br>Milk kg (200d LA1)	6:61,694,163-61,752,222	*GRXCR1*	6:61,233,082-62,233,082	*ENSBTAG00000054299, ATP8A1, ENSBTAG00000051484*
6:62,988,117<br>6:63,010,380	Milk kg (LA1),<br>Milk kg (200d LA1)	6:62,798,692-63,092,317	*KCTD8*	6:62,488,117-63,510,380	*ENSBTAG00000055052, YIPF7, GUF1, GNPDA2*
6:64,928,624	Milk kg (200d LA1)	6:64,927,963-65,016,132	*GABRA2*	6:64,428,624-65,428,624	*GABRG1, COX7B2, H4C14*
6:77,688,509	Milk kg (LA1),<br>Milk kg (200d LA1)	6:77,603,159-77,688,509	*ADGRL3*	6:77,188,509-78,188,509	*ENSBTAG00000053291*
6:80,530,130<br>6:80,626,467	Milk kg (LA1),<br>Milk kg (100d LA1),<br>Milk kg (200d LA1)<br>Milk kg (100d LA1),<br>Milk kg (200d LA1)	6:80,530,130-80,626,467	-	6:80,030,130-81,126,467	*ENSBTAG00000050977, EPHA5, ENSBTAG00000053125*
6:86,112,142	Milk kg (LA1),<br>Milk kg (100d LA1),<br>Milk kg (200d LA1),<br>Protein kg (100d LA1)	6:85,633,295-87,011,619	*CSN3, CABS1, AMTN, AMBN, ENAM, JCHAIN, ENSBTAG00000055134, UTP3, RUFY3, GRSF1, ENSBTAG00000051236, MOB1B, DCK, SLC4A4, GC, ENSBTAG00000049290*	6:85,612,142-86,612,142	-

6:87,266,808	Protein kg (100d LA1)	6:87,156,735-87,266,808	*NPFFR2*	6:86,766,808-87,766,808	*SLC4A4, GC, ENSBTAG00000049290, ADAMTS3*
6:88,164,411	Protein kg (200d LA1)	6:88,024,804-88,412,357	*COX18, ANKRD17*	6:87,664,411-88,664,411	*ADAMTS3, ALB, AFP, AFM, ENSBTAG00000049436, RASSF6*
8:53,663,120	Milk kg (LA3), Milk kg (LAm)	8:53,467,317-54,449,954	*GNA14, GNAQ, CEP78, PSAT1*	8:53,163,120-54,367,972	*ENSBTAG00000051821, ENSBTAG00000017734*
8:53,867,972	Milk kg (LA3)				
8:59,101,606	Milk kg (LAm), Protein kg (LAm)	8:58,948,243-59,431,157	*ENSBTAG00000049991, FAM205C, PHF24, DNAJB5, ENSBTAG00000051343, VCP, FANCG, PIGO, STOML2, FAM214B, UNC13B*	8:58,601,606-59,601,606	-
8:100,876,785	Fat kg (LAm)	8:100,456,448-100,905,954	*LPAR1, OR2K2, ECPAS*	8:100,376,785-101,376,785	*ZNF483, PTGR1, DNAJC25, GNG10, SHOC1, UGCG*
9:10,638,013	Milk kg (LA3), Milk kg (LAm)	9:10,632,814-10,713,987	*ENSBTAG00000048046*	9:10,138,013-11,138,013	*ENSBTAG00000053784, OGFRL1, RIMS1*
9:26,353,699	Fat kg (LA1)	9:26,317,865-26,353,699	-	9:25,853,699-26,853,699	*HDDC2, TPD52L1, RNF217, NKAIN2, ENSBTAG00000051569*
10:3,611,602	Fat % (200d LA1)	10:3,582,722-3,611,602	-	10:3,111,602-4,111,602	*ENSBTAG00000052874, KCNN2, ENSBTAG00000023186, ENSBTAG00000053655, TRIM36, PGGT1B, CCDC112*
11:69,443,503	Protein kg (LA2)	11:69,325,299-69,497,386	*LCLAT1*	11:68,943,503-69,943,503	*GALNT14, CAPN13, LBH, YPEL5*
11:92,712,210	Fat % (200d LA1)	11:92,712,210-92,931,923	*ENSBTAG00000012827*	11:92,212,210-93,212,210	*ENSBTAG00000039201, ENSBTAG00000039186, GGTA1, DAB2IP, NDUFA8, MORN5, LHX6, RBM18, MRRF*
12:66,340,756	Fat % (200d LA1)	12:66,340,756-66,475,106	*GPC5*	12:65,840,756-66,840,756	-
14:26,340,400	Protein kg (100d LA1)	14:26,125,048-26,369,536	*RAB2A, CHD7*	14:25,840,400-26,840,400	*CA8*
16:11,516,923	Fat % (200d LA1)	16:11,446,923-11,586,923	-	16:11,016,923-12,016,923	-
16:30,668,830	Fat % (LA3)	16:30,668,830-30,855,245	*H3-5, ENSBTAG00000021109, CNST, TFB2M, SMYD3*	16:30,168,830-31,168,830	*CDC42BPA, AHCTF1, ENSBTAG00000049714, SCCPDH*

16:32,105,683	Fat % (200d LA1)	16:32,035,645-32,105,683	-	16:31,605,683-32,605,683	*KIF26B, EFCAB2, ENSBTAG00000047437, HNRNPU, COX20, DESI2, ENSBTAG00000044066*
16:33,789,714	Fat % (LA2), Fat % (200d LA1)	16:33,712,006-33,789,714	*SDCCAG8*	16:33,289,714-34,289,714	*AKT3, CEP170*
16:40,391,486	Fat % (200d LA1)	16:40,327,167-40,793,811	*TNFSF18, ENSBTAG00000052047, ENSBTAG00000053302*	16:39,891,486-40,891,486	*DNM3, C16H1orf105, PIGC, SUCO, FASLG, ENSBTAG00000016347, TNFSF4, ENSBTAG00000020550*
16:46,683,276	Protein kg (100d LA1)	16:46,250,903-46,683,276	*ENSBTAG00000054938*	16:46,183,276-47,273,225	*DNAJC11, ENSBTAG00000049238, THAP3, PHF13, KLHL21, ZBTB48, TAS1R1, NOL9, PLEKHG5, TNFRSF25, ESPN, HES2, ACOT7, GPR153, HES3, ENSBTAG00000048433, ICMT, RNF207, RPL22, CHD5 , KCNAB2*
16:46,773,225	Protein kg (100d LA1)	16:46,712,441-47,068,054	*DNAJC11, ENSBTAG00000049238, THAP3, PHF13, KLHL21, ZBTB48, TAS1R1, NOL9, PLEKHG5, TNFRSF25, ESPN, HES2, ACOT7*		
18:53,596,284	Fat % (LA2)	18:53,340,459-53,596,284	*RSPH6A, SYMPK, FOXA3, MYPOP, ENSBTAG00000050077, ENSBTAG00000054722, NANOS2, NOVA2, CCDC61, PGLYRP1, ENSBTAG00000023367, IGFL1, HIF3A, PPP5C*	18:53,096,284-54,096,284	*PPM1N, VASP, ENSBTAG00000049026, OPA3, GPR4, EML2, GIPR, SNRPD2, QPCTL, FBXO46, SIX5, DMPK, DMWD, PNMA8A, CCDC8, CALM3, PTGIR, GNG8, DACT3, ENSBTAG00000052862, PRKD2, STRN4, FKRP, SLC1A5, AP2S1, ARHGAP35, NPAS1, TMEM160*
20:50,879,180	Fat % (200d LA1)	20:50,585,802-50,879,180	-	20:50,379,180-51,379,180	*CDH12*
20:71,448,297	Milk kg (LA2), Protein kg (LA2), Fat kg (LA2)	20:71,378,297-71,518,297	*TRIP13, BRD9, ENSBTAG00000054687, TPPP, CEP72*	20:70,948,297-71,948,297	*LPCAT1, SLC6A3, CLPTM1L, TERT, SLC6A18, SLC6A19, ENSBTAG00000049547, SLC12A7, NKD2, SLC9A3, EXOC3, AHRR, PDCD6, SDHA, LRRC14B, CCDC127, ENSBTAG00000055240, ENSBTAG00000048135, ENSBTAG00000047700, ENSBTAG00000047632*
21:42,828,439	Protein kg (200d LA1)	21:42,789,940-42,828,439	*AKAP6*	21:42,328,439-43,328,439	*NUBPL, ENSBTAG00000055201, ARHGAP5*
24:16,789,760	Milk kg (LA3)	24:16,717,203-16,839,012	-	24:16,289,760-17,289,760	*ENSBTAG00000049926*

24:30,250,034	Protein kg (LA1), Protein kg (200d LA1), Fat kg (LA1)	24:30,129,550-30,250,034	-	24:29,750,034-30,750,034	*CHST9, AQP4, KCTD1, TAF4B, PSMA8, SS18*
25:7,944,597	Fat % (200d LA1)	25:7,944,597-8,013,450	-	25:7,444,597-8,444,597	*TMEM114, METTL22, ABAT, TMEM186, PMM2, CARHSP1, USP7, C25H16orf72*
25:9,711,895	Fat kg (LA1), Fat kg (200d LA1)	25:9,684,634-9,736,499	*CLEC16A*	25:9,211,895-10,211,895	*ATF7IP2, EMP2, TEKT5, NUBP1, TVP23A, CIITA, DEXI, SOCS1, TNP2, PRM3, PRM2, PRM1, RMI2, ENSBTAG00000013305*
25:11,019,450	Milk kg (200d LA1)	25:10,978,440-11,040,225	*SNX29*	25:10,519,450-11,519,450	*BCAR4, RSL1D1, GSPT1, ENSBTAG00000053512, TNFRSF17, ENSBTAG00000051973, CPPED1*
27:13,864,569	Fat % (LA2), Fat % (200d LA1)	27:13,806,128-13,945,896	*DCTD*	27:13,364,569-143,64,569	*ENSBTAG00000047749, TENM3, WWC2, ENSBTAG00000052405, ENSBTAG00000051927, CDKN2AIP, ING2*
27:18,527,112	Fat kg (LA1)	27:18,417,234-18,527,112	*ENSBTAG00000047159*	27:18,027,112-19,027,112	*ENSBTAG00000005559*
28:25,196,334	Protein kg (100d LA1)	28:25,126,334-25,266,334	*STOX1, DDX50, DDX21, KIFBP*	28:24,696,334-25,696,334	*PBLD, HNRNPH3, RUFY2, DNA2, SLC25A16, TET1, CCAR1, SRGN, VPS26A, SUPV3L1, HKDC1, HK1*
29:50,217,955	Fat % (LA2)	29:49,953,631-50,217,955	*DUSP8, MOB2, TOLLIP, ENSBTAG00000052368, ENSBTAG00000054135, ENSBTAG00000050966, ENSBTAG00000052981, ENSBTAG00000008274*	29:49,717,955-50,826,170	*CTSD, IFITM10, ENSBTAG00000019579, ENSBTAG00000054020, ENSBTAG00000050212, AP2A2, CHID1, TSPAN4, POLR2L, CD151, CRACR2B, PNPLA2, RPLP2, PIDD1, SLC25A22, CEND1, GATD1, TALDO1, EPS8L2, TMEM80, DEAF1, DRD4, ENSBTAG00000054832, CDHR5, IRF7, PHRF1, RASSF7, LMNTD2, LRRC56, HRAS, RNH1, PTDSS2*
29:50,260,533	Fat % (LA2)	29:50,229,562-50,326,170	*ENSBTAG00000050398*		
29:50,326,170	Fat % (LA2)				
X:12,896,716	Milk kg (LA3)	X:12,401,670-13,349,470	-	X:12,396,716-13,396,716	
X:116,886,837	Protein kg (200d LA1)	X:116,886,837-116,908,031	-	X:116,386,837-118,191,901	*PPP4R3C, ENSBTAG00000053213, ENSBTAG00000018583, ENSBTAG00000053624, ENSBTAG00000048848*
X:117,691,901	Protein kg (LA3), Protein kg (LAm)	X:117,668,729-117,691,901	-		
X:133,244,405	Fat kg (200d LA1)	X:133,174,405-133,314,405	*SHROOM2, GPR143*	X:132,744,405-133,744,405	*ENSBTAG00000048594, ENSBTAG00000035122, ENSBTAG00000009932, ENSBTAG00000055193, CLDN34, ENSBTAG00000053828, ENSBTAG00000047068, ENSBTAG00000002158, ENSBTAG00000051024, TBL1X*

Supplementary Table 9: List of genes located in the haplotype or within 1 Mb of the respective SNP in DSN for protein content with a p<0.0005 (Supplementary Table 4). Haplotype blocks are listed as chromosome:start:stop in bp together with the SNPs, the associated trait and the genes falling into the haplotype blocks. Genes are listed with their gene name or Ensembl ID if no gene name was available. If consecutive haplotype or 1 Mb gene regions overlapped, they were merged together.

**Significant SNP**	**Traits**	**Haplotype region**	**Genes in haplotype region**	**1Mb region**	**Additional genes in 1 Mb region**
2:128,295,010	Protein % (LA2)	2:128,295,010-128,377,966	*NCMAP*	2:127,795,010-128,795,010	*RUNX3, CLIC4, ENSBTAG00000054129, SRRM1, RCAN3, NIPAL3, STPG1, GRHL3, IFNLR1, IL22RA1, MYOM3*
3:15,470,670	Protein % (LA2)	3:15,089,971-15,762,364	*DAP3, ASH1L, RUSC1, FDPS, PKLR, HCN3, CLK2, SCAMP3, FAM189B, GBA, MTX1, THBS3, MUC1, TRIM46, KRTCAP2, DPM3, SLC50A1, EFNA1, EFNA3, EFNA4, ADAM15, DCST1, ZBTB7B, FLAD1, CKS1B, SHC1, PBXIP1, PMVK, KCNN3*	3:14,970,670-15,970,670	*GON4L, MSTO1, ADAR*
3:21,692,628	Protein % (LA1), Protein % (200d LA1)	3:21,576,250-21,692,628	*RNF115, ENSBTAG00000053114, PDZK1*	3:21,192,628-22,192,628	*HJV, TXNIP, POLR3GL, ANKRD34A, RBM8A, ENSBTAG00000008370, PEX11B, ITGA10, ANKRD35, BCL9, ENSBTAG00000052421, PIAS3, NUDT17, POLR3C, GPR89A, GJA8, GJA5, ACP6*
3:21,764,453	Protein % (LA1), Protein % (LA2), Protein % (LAm), Protein % (200d LA1)	3:21,764,453-21,819,709	-	3:21,264,453-22,264,453	*HJV, TXNIP, POLR3GL, ANKRD34A, RBM8A, ENSBTAG00000008370, PEX11B, ITGA10, ANKRD35, ENSBTAG00000052421, PIAS3, NUDT17, POLR3C, RNF115, ENSBTAG00000053114, PDZK1, GPR89A, GJA8, GJA5, ACP6, BCL9, ENSBTAG00000046242, ENSBTAG00000046872*
3:26,278,860	Protein % (LA1)	3:26,126,232-26,278,860	*TTF2, CD101, PTGFRN*	3:25,778,860-26,778,860	*MAN1A2, VTCN1, TRIM45, CD2, IGSF3, CD58*
5:74,853,402	Protein % (LA1), Protein % (200d LA1)	5:74,783,402-74,923,402	*MYH9, TXN2, FOXRED2*	5:74,353,402-75,353,402	*ENSBTAG00000052884, CSF2RB, ENSBTAG00000055135, NCF4, ENSBTAG00000048740, PVALB, ENSBTAG00000050417, IFT27, ENSBTAG00000039524, ENSBTAG00000038652, APOL3, ENSBTAG00000012192, EIF3D,*

					ENSBTAG00000054427, CACNG2, ENSBTAG00000008137
6:65,962,733	Protein % (LA2)	6:65,801,992-66,039,725	*GABRB1*	6:65,462,733-66,462,733	*GABRA4, COMMD8, ATP10D, CORIN, ENSBTAG00000053278*
6:80,530,130	Protein % (LA1), Protein % (100d LA1), Protein % (200d LA1)	6:80,530,130-80,626,467	-	6:80,030,130-81,030,130	*ENSBTAG00000050977, EPHA5*
10:34,947,852	Protein % (LA2), Protein % (200d LA1)	10:34,877,852-35,017,852	-	10:34,447,852-35,447,852	*THBS1, FSIP1*
10:35,164,676	Protein % (LA2)	10:34,282,362-36,552,953	*THBS1, FSIP1, GPR176, INO80, DLL4, ENSBTAG00000054650, EIF2AK4, SRP14, BMF, BUB1B, PAK6, ANKRD63, PLCB2, INAFM2, CCDC9B, PHGR1, DISP2, KNSTRN, IVD, BAHD1, CHST14, CCDC32, RPUSD2, KNL1, VPS18, CHAC1, ENSBTAG00000048559, RAD51, RMDN3, GCHFR, DNAJC17, C10H15orf62, ZFYVE19, PPP1R14D, SPINT1, RHOV*	10:34,664,676-35,664,676	-
10:44,773,979	Protein % (LA2)	10:44,505,055-44,773,979	*FRMD6, GNG2*	10:44,273,979-45,273,979	*RTRAF, NID2, ENSBTAG00000001423, PTGDR, PLEKHO2, PIF1*
10:46,450,562	Protein % (LA2)	10:46,330,098-46,672,954	*ENSBTAG00000054388, CA12, ENSBTAG00000050908, APH1B, ENSBTAG00000019474, FBXL22, USP3,*	10:45,950,562-46,950,562	*DAPK2, HERC1, ENSBTAG00000052707, ENSBTAG00000051076, RAB8B, RPS27L, LACTB*
10:47,670,717	Protein % (100d LA1)	10:47,550,105-47,729,354	*TLN2*	10:47,170,717-48,170,717	*C2CD4B, ENSBTAG00000010952, VPS13C*
18:33,540,904	Protein % (LA2)	18:33,470,904-33,610,904	-	18:33,040,904-34,040,904	-
20:50,879,180	Protein % (LA1), Protein % (LA2), Protein % (200d LA1), Fat % (LA1), Fat % (200d LA1)	20:50,585,802-50,879,180	-	20:50,379,180-51,379,180	*CDH12*
28:18,458,637	Protein % (LA1)	28:18,369,569-18,458,637	*RTKN2*	28:17,958,637-18,958,637	*ARID5B, ENSBTAG00000040018, ZNF365, ADO, EGR2*

1.8 Manhattan plots

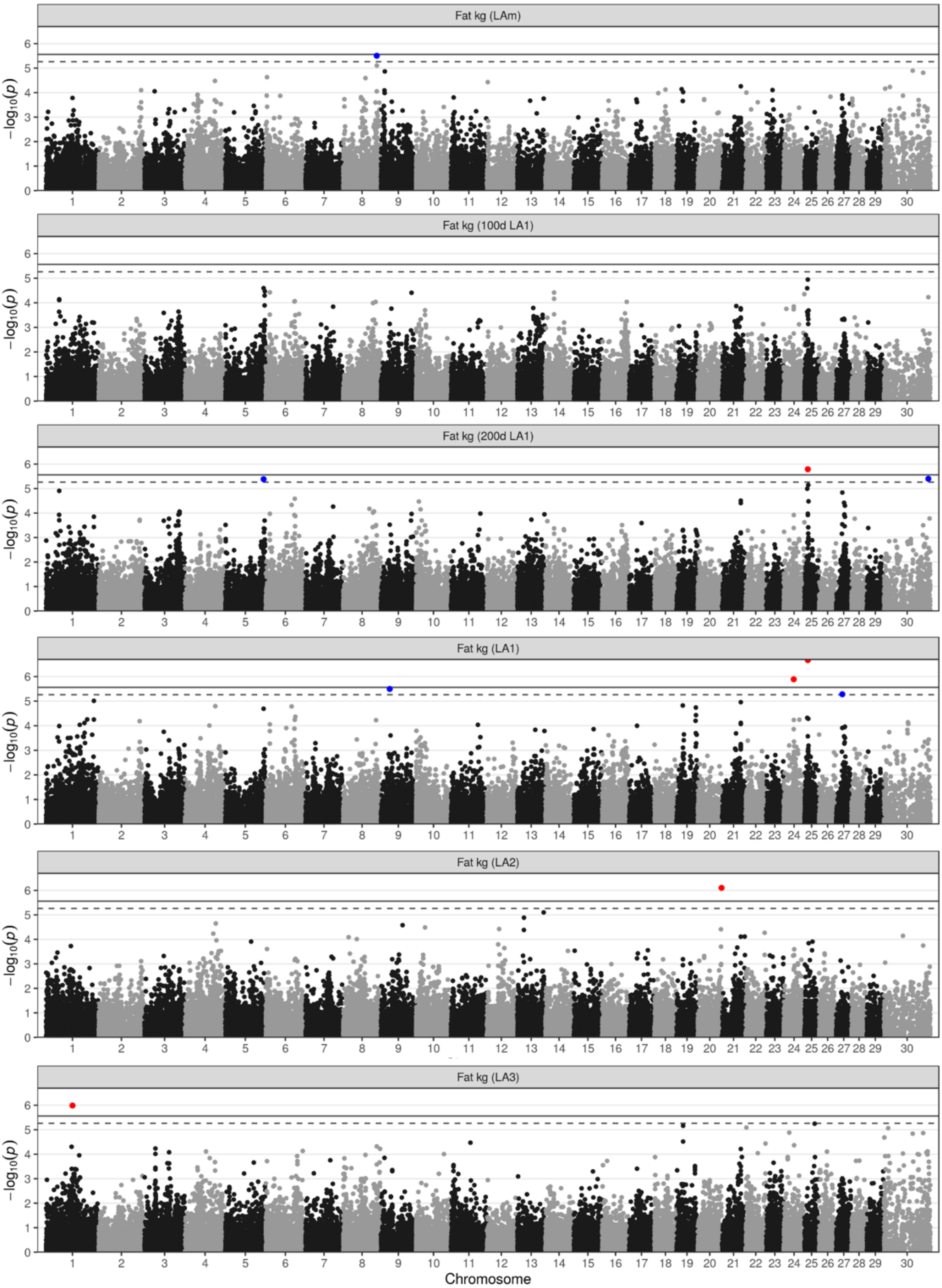

Supplementary Figure 4: Manhattan plots for fat yield in kg. Plots are shown for the lactation mean of the 305d performance (LAm), the 100d and 200d performance in LA1, and the 305-days performance in the first three lactations (LA1-LA3). Markers above the significance or suggestive thresholds are highlighted in red (solid line, $\alpha < 0.05$) or blue (dashed line, $\alpha < 0.1$), respectively.

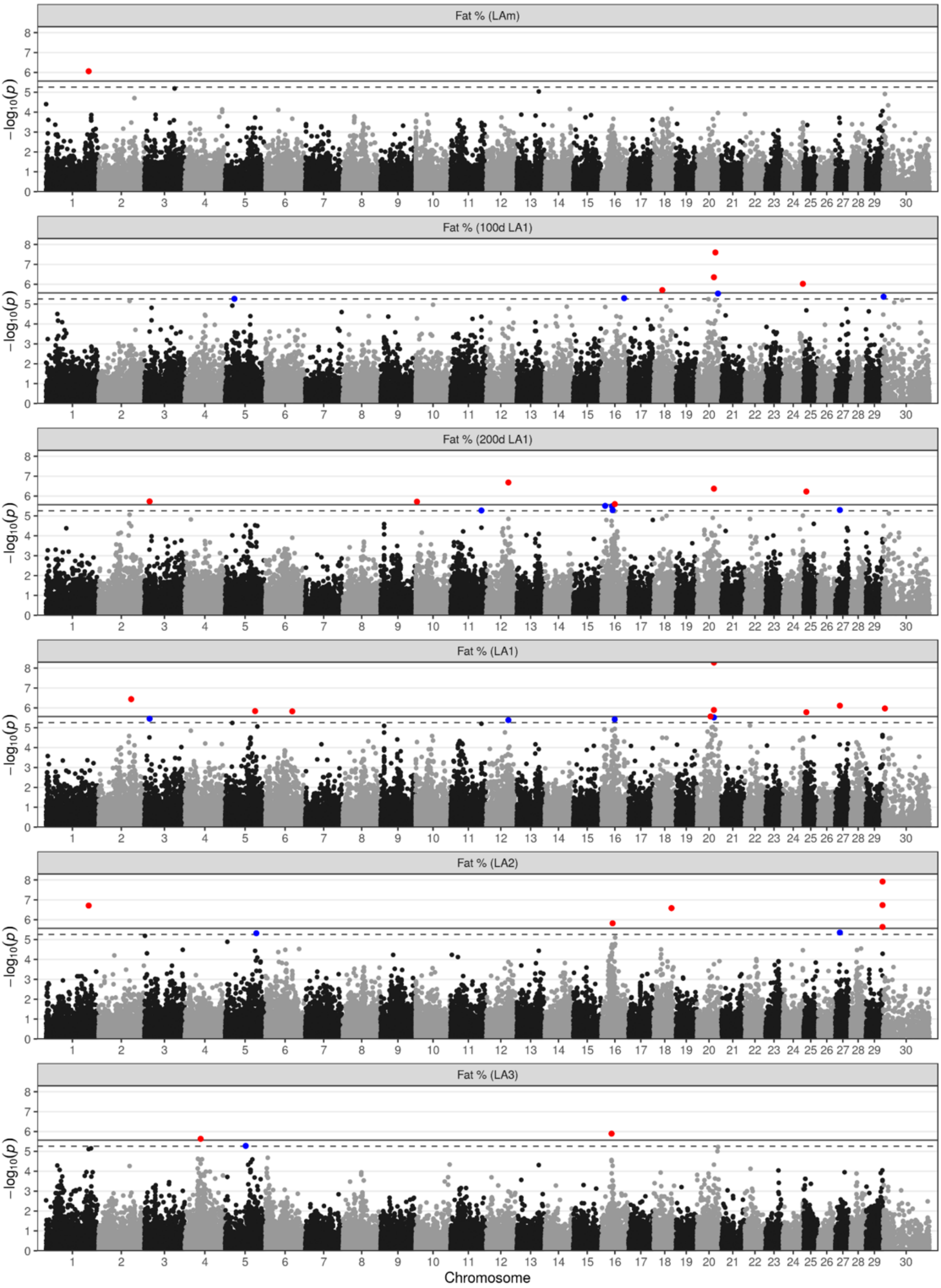

Supplementary Figure 5: Manhattan plots for fat content in %. Plots are shown for the lactation mean of the 305d performance (LAm), the 100d and 200d performance in LA1, and the 305-days performance in the first three lactations (LA1-LA3). Markers above the significance or suggestive thresholds are highlighted in red (solid line, $\alpha < 0.05$) or blue (dashed line, $\alpha < 0.1$), respectively.

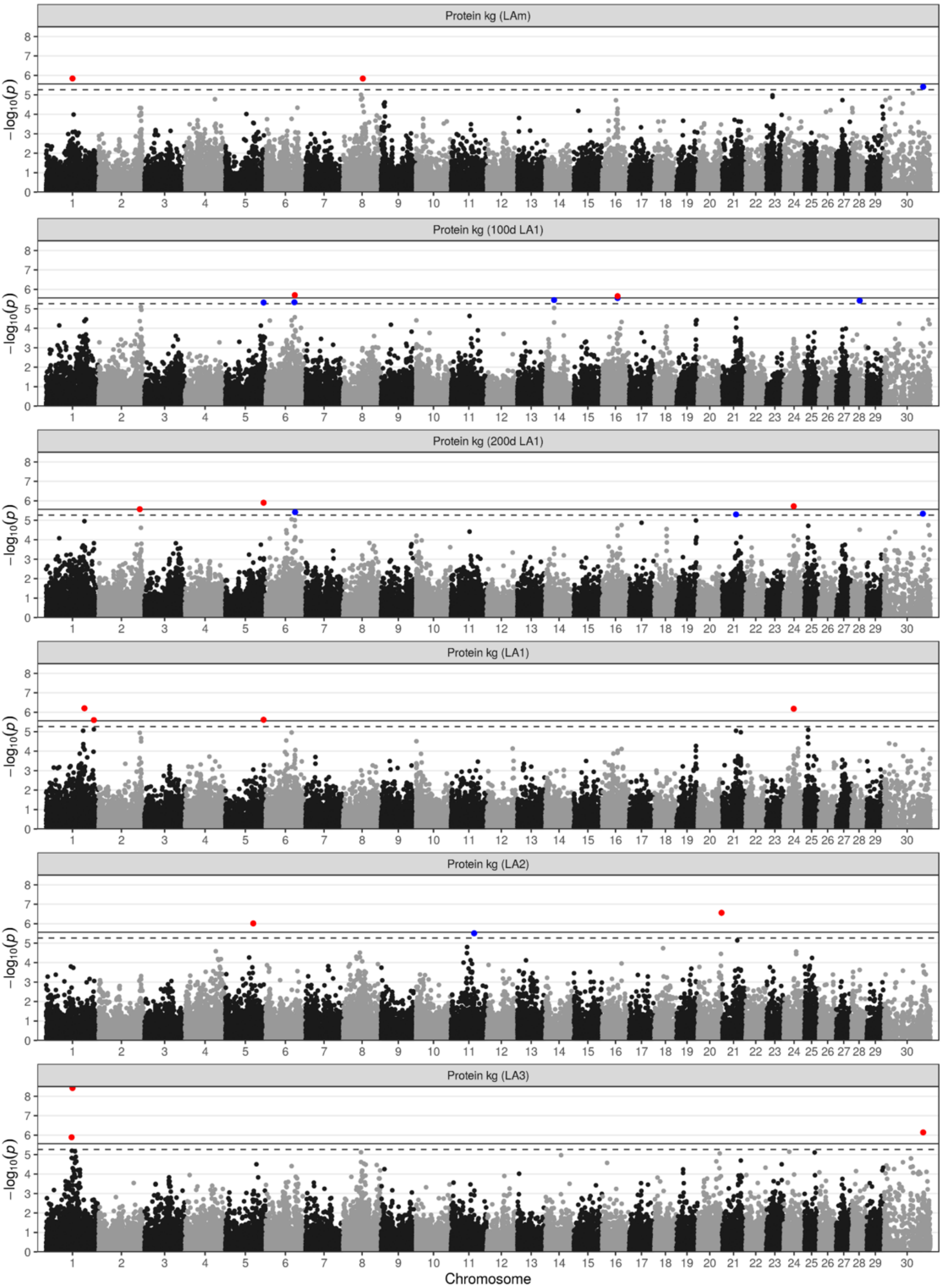

Supplementary Figure 6: Manhattan plots for protein yield in kg. Plots are shown for the lactation mean of the 305d performance (LAm), the 100d and 200d performance in LA1, and the 305-days performance in the first three lactations (LA1-LA3). Markers above the significance or suggestive thresholds are highlighted in red (solid line, $\alpha < 0.05$) or blue (dashed line, $\alpha < 0.1$), respectively.

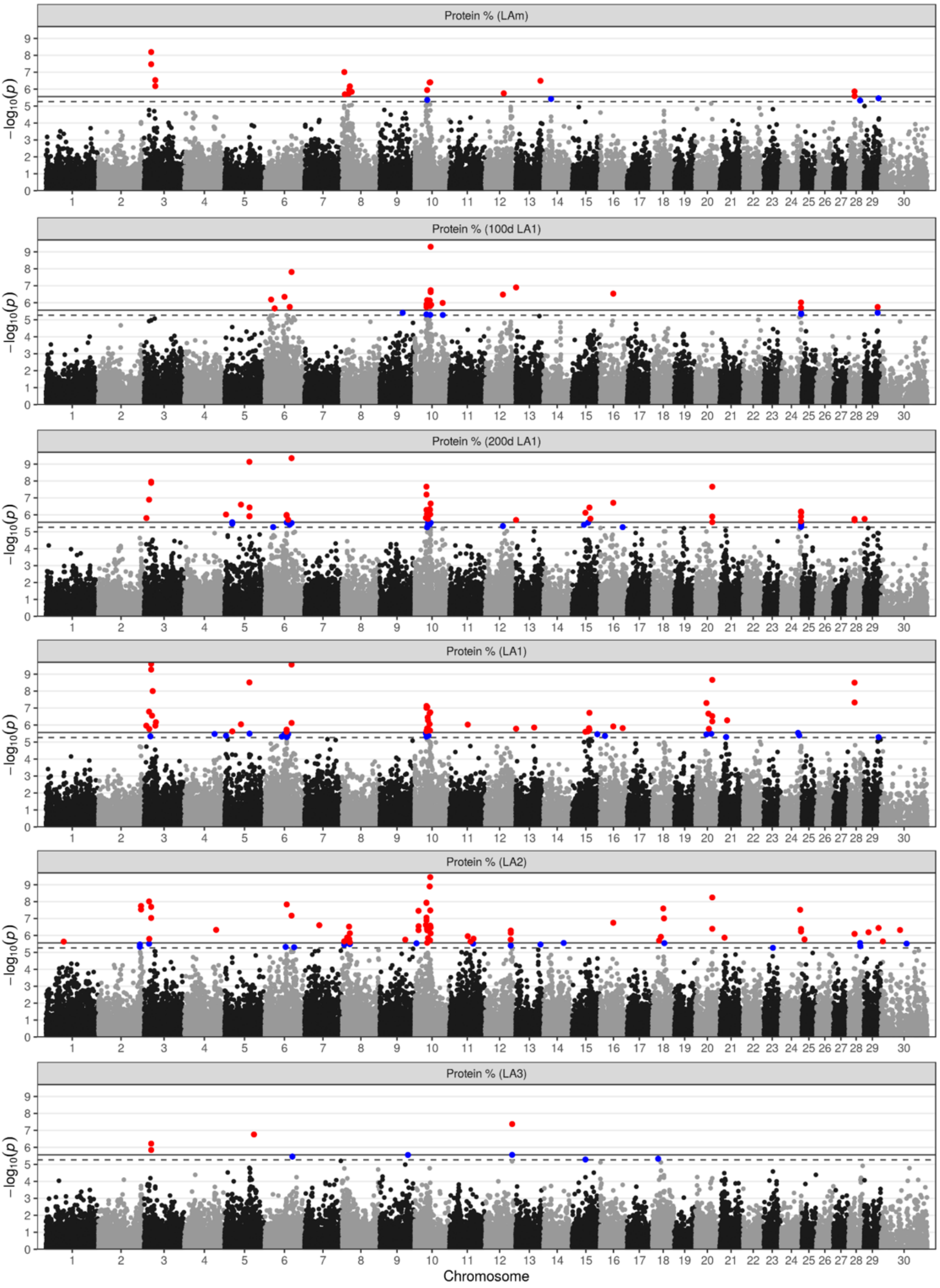

Supplementary Figure 7: Manhattan plots for protein content in %. Plots are shown for the lactation mean of the 305d performance (LAm), the 100d and 200d performance in LA1, and the 305-days performance in the first three lactations (LA1-LA3). Markers above the significance or suggestive thresholds are highlighted in red (solid line, $\alpha < 0.05$) or blue (dashed line, $\alpha < 0.1$), respectively.

1.9 Comparison to significant SNPs for milk production traits in Holstein

Supplementary Table 10: SNPs associated with milk production traits in German Holstein compared to DSN. SNPs that were significantly associated with milk production traits in Holstein bulls were filtered to be located <500kb to candidate milk production genes in cattle (Ogorevc et.al, 2009). Respective genes are listed with their chromosome (Chr), gene start and stop, the associated SNP in Holstein, the reference (Ref) and alternative allele (Alt), the significantly associated breeding value, the favorable allele (FA) in Holstein, and the FA frequency (FAF) of Holstein and DSN. The higher FAF is highlighted in green.

Gene name	Chr	Gene start (bp)	Gene stop (bp)	SNP (chr:bp)	Ref	Alt	Breeding value	FA (Holstein)	FAF (Holstein)	FAF (DSN)
ABCG2	6	36,475,377	36,603,209	6:36,773,960	G	A	Milk kg	G	0.56	0.84
CSN1S1	6	85,411,118	85,429,268	6:85,092,523	A	G	Protein %	A	0.38	0.84
				6:85,067,620	A	G	Protein %	A	0.35	0.70
CSN1S2	6	85,529,905	85,548,556	6:85,092,523	A	G	Protein %	A	0.38	0.84
				6:85,067,620	A	G	Protein %	A	0.35	0.70
CSN2	6	85,449,164	85,457,744	6:85,092,523	A	G	Protein %	A	0.38	0.84
				6:85,067,620	A	G	Protein %	A	0.35	0.70
DGAT1	14	603,813	612,791	14:305,780	C	T	Milk kg	C	0.56	0.62
							Fat kg	T	0.44	0.38
							Protein kg	C	0.56	0.62
							Fat %	T	0.44	0.38
							Protein %	T	0.44	0.38
				14:490,055	A	G	Milk kg	A	0.53	0.93
							Fat kg	G	0.47	0.07
							Protein kg	A	0.53	0.93
							Fat %	G	0.47	0.07
							Protein %	G	0.47	0.07
				14:511,247	C	A	Milk kg	A	0.53	0.83
							Fat kg	C	0.47	0.17
							Protein kg	A	0.53	0.83
							Fat %	C	0.47	0.17
							Protein %	C	0.47	0.17
GHR	20	31,868,624	32,178,311	20:32,009,781	T	C	Protein %	T	0.72	0.26
				20:31,532,284	C	T	Protein %	T	0.63	0.50
GPAT4	27	36,522,605	36,539,773	27:36,466,414	C	T	Fat %	C	0.65	0.87
PAEP	11	103,255,824	103,264,276	11:103,120,186	C	T	Milk kg	C	0.73	0.75
							Protein kg	C	0.73	0.75
				11:103,068,347	G	A	Milk kg	G	0.73	0.77
							Protein kg	G	0.73	0.77
				11:103,243,510	G	A	Milk kg	A	0.45	0.54
							Protein kg	A	0.45	0.54
PRLR	20	38,971,752	39,100,563	20:38,885,278	G	A	Protein %	A	0.82	0.44
				20:39,445,232	C	T	Protein %	T	0.75	0.58
SPP1	6	36,693,193	36,730,559	6:36,773,960	G	A	Milk kg	G	0.56	0.84

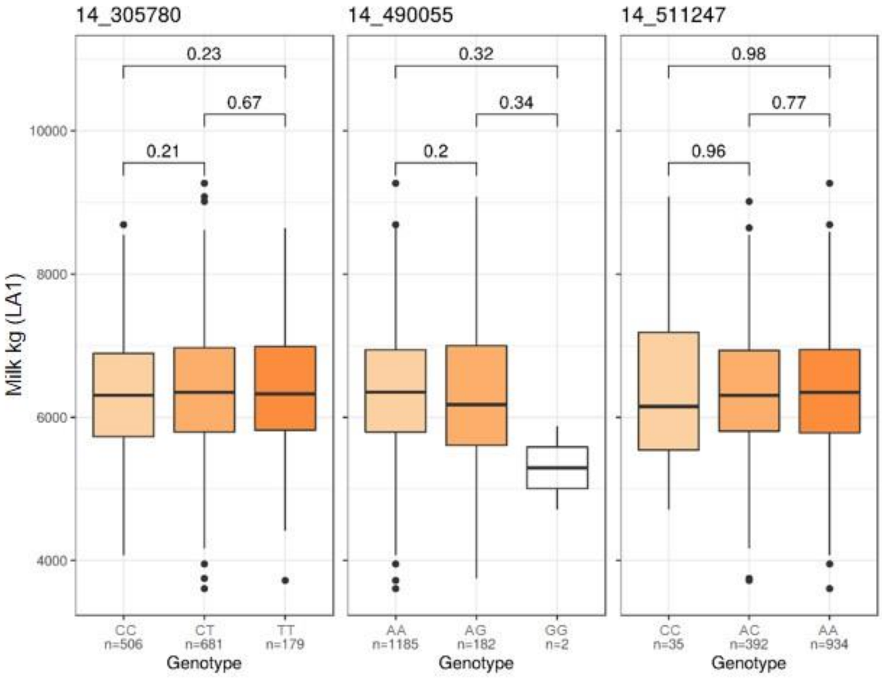

Supplementary Figure 8: SNP effect plots for milk yield in LA1 of three SNPs closest to *DGAT1* gene. No significance was found in DSN. Genotype groups with less than 30 observations were not used in order to prevent spurious associations, thus they are only shown for the sake of completeness (white boxplots).

1.10 Comparison to publications

Supplementary Table 11: Comparison of identified SNPs to other publications. The identified SNPs are listed together with their SNP-ID, the associated trait in this study, the associated trait of the corresponding publication, and the breed definition from the publication. SNPs were highlighted in green, if the associated trait in this study was the same as found in another publication, or highlighted in yellow, if the associated trait of the other publication was another milk trait.

SNP, rsID	Associated trait	Associated trait (publication)	Breed (publication)
1:128,503,005, rs109686415	Fat % (LA2), Fat % (LAm)	Lean meat yield (Doran et al., 2014)	Holstein
3:15,470,670, rs110073735	Protein % (LA2)	Milk yield (Jiang et al., 2019)	Holstein
		Milk protein % (Jiang et al., 2019; Nayeri et al., 2016)	Holstein
		Milk fat % (Jiang et al., 2019)	Holstein
		Milk lactose % (Costa et al., 2019)	Fleckvieh
3:21,692,628, rs41587408	Protein % (LA1), Protein % (200d LA1)	Milk protein % (Kolbehdari et al., 2009)	Holstein
		Milk fat % (Kolbehdari et al., 2009)	
3:21,764,453, rs110474631	Protein % (LA1), Protein % (LA2), Protein % (LAm), Protein % (200d LA1)	Body weight gain (Snelling et al., 2010)	Charolais, Gelbvieh, Hereford, Limousin, Pinzgauer, Red Angus, Red Poll, Simmental, Angus
5:74,853,402, rs110803736	Protein % (LA1), Protein % (200d LA1)	Milk fat yield (Jiang et al., 2019)	Holstein
		Conception rate (Jiang et al., 2019)	Holstein
6:64,928,624, rs42482917	Milk kg (200d LA1)	Milk kappa-casein percentage (Buitenhuis et al., 2016)	Holstein
6:65,962,733, rs42224984	Protein % (LA2)	Milk unglycosylated kappa-casein % (Buitenhuis et al., 2016)	Holstein
		Milk glycosylated kappa-casein % (Buitenhuis et al., 2016)	

		Milk kappa-casein % (Buitenhuis et al., 2016)	
6:77,688,509, rs41652041	Milk kg (LA1), Milk kg (200d LA1)	Milk protein % (Jiang et al., 2019)	Holstein
6:80,530,130, rs110291935	Milk kg (LA1), Milk kg (100d LA1), Milk kg (200d LA1), Protein % (LA1), Protein % (100d LA1), Protein % (200d LA1)	Curd firmness (Dadousis et al., 2017)	Brown Swiss
		Milk kappa-casein % (Buitenhuis et al., 2016)	Holstein
		Milk unglycosylated kappa-casein % (Buitenhuis et al., 2016)	Holstein
		Milk glycosylated kappa-casein % (Buitenhuis et al., 2016)	Holstein
		Cheese fat recovery (Dadousis et al., 2017)	Brown Swiss
6:80,626,467, rs109872424	Milk kg (100d LA1), Milk kg (200d LA1)	Curd firmness (Dadousis et al., 2017)	Brown Swiss
		Milk glycosylated kappa-casein % (Buitenhuis et al., 2016)	Holstein
		Milk kappa-casein % (Buitenhuis et al., 2016)	Holstein
		Milk unglycosylated kappa-casein % (Buitenhuis et al., 2016)	Holstein
		Cheese fat recovery (Dadousis et al., 2017)	Brown Swiss
6:86,112,142, rs109592101	Milk kg (LA1), Milk kg (100d LA1), Milk kg (200d LA1), Protein kg (100d LA1)	Facial pigmentation (Mészáros et al., 2015)	Fleckvieh
		Eye area pigmentation (Mészáros et al., 2015)	Fleckvieh
		Milk kappa-casein % (Buitenhuis et al., 2016)	Holstein
		Milk glycosylated kappa-casein % (Buitenhuis et al., 2016)	Holstein
		Milk unglycosylated kappa-casein % (Buitenhuis et al., 2016)	Holstein
		Somatic cell score (Jiang et al., 2019)	Holstein
		Milk yield (Jiang et al., 2019)	Holstein
		Milk protein yield (Jiang et al., 2019)	Holstein
6:87,266,808, rs41591365	Protein kg (100d LA1)	Milk protein yield (Jiang et al., 2019; Meredith et al., 2012)	Holstein
		Ketosis (Nayeri et al., 2019)	Holstein
6:88,164,411, rs41622837	Protein kg (200d LA1)	Daughter pregnancy rate (Parker Gaddis et al., 2016)	Holstein
8:53,663,120, rs41793393	Milk kg (LA3), Milk kg (LAm)	Rump angle (Cole et al., 2011)	Holstein
		Milk riboflavin content (Poulsen et al., 2015)	Holstein
10:34,947,852, rs41601192	Protein % (LA2), Protein % (200d LA1)	Age at puberty (Hawken et al., 2012)	Brahman
10:35,164,676, rs29016462	Protein % (LA2)	Muscle anserine content (Mateescu et al., 2017)	Angus
10:46,450,562, rs109605174	Protein % (LA2)	Milk protein % (Jiang et al., 2019; Wang et al., 2019)	Holstein
11:92,712,210, rs110540697	Fat % (200d LA1)	Tick resistance (Mapholi et al., 2016)	Nguni
16:40,391,486, rs41804404	Fat % (200d LA1)	Intramuscular fat (Bolormaa et al., 2011)	Brahman, Hereford, Angus
16:40,391,486, rs41804404	Fat % (200d LA1)	Milk protein yield (Jiang et al., 2019)	Holstein
18:33,540,904, rs41869985	Protein % (LA2)	Polyunsaturated fatty acid content (Mateescu et al., 2017)	Angus
18:53,596,284, rs109907036	Fat % (LA2)	Calving interval (Aliloo et al., 2015)	jersey
20:50,879,180, rs41948928	Protein % (LA1), Protein % (LA2), Protein % (200d LA1), Fat % (LA1),	Length of productive life (Cole et al., 2011)	Holstein
		Milk protein % (Cole et al., 2011)	Holstein
		Udder depth (Cole et al., 2011)	Holstein
		Stillbirth (maternal) (Cole et al., 2011)	Holstein
		Milk fat yield (Cole et al., 2011)	Holstein

	Fat % (200d LA1)	Milk protein yield (Cole et al., 2011)	Holstein
		Daughter pregnancy rate (Cole et al., 2011)	Holstein
20:71,448,297, rs110353352	Milk kg (LA2), Protein kg (LA2), Fat kg (LA2)	Longissimus muscle area (Li et al., 2017)	Hanwoo
25:7,944,597, rs109583598	Fat % (200d LA1)	Marbling score (Li et al., 2017)	Hanwoo
25:11,019,450, rs109027867	Milk kg (200d LA1)	Intramuscular fat (Mateescu et al., 2017)	Angus
28:25,196,334, rs41587054	Protein kg (100d LA1)	Body weight (yearling) (Snelling et al., 2010)	Charolais, Gelbvieh, Hereford, Limousin, Pinzgauer, Red Angus, Red Poll, Simmental, Angus
29:50,217,955, rs109241029	Fat % (LA2)	Body weight gain (Snelling et al., 2010)	
		Body weight (yearling) (Snelling et al., 2010)	
29:50,260,533, rs109840529	Fat % (LA2)	Body weight gain (Snelling et al., 2010)	
X:12,896,716, rs109188619	Milk kg (LA3)	Udder attachment (Cole et al., 2011)	Holstein
		Milk fat yield (Cole et al., 2011)	
		Feet and leg conformation (Cole et al., 2011)	
		Milk fat % (Cole et al., 2011)	
		Teat placement – front (Cole et al., 2011)	
		Milk yield (Cole et al., 2011)	
		Net merit (Cole et al., 2011)	
		Milk protein % (Cole et al., 2011)	
		Milk protein yield (Cole et al., 2011)	
		Body depth (Cole et al., 2011)	
		Foot angle (Cole et al., 2011)	
		Udder height (Cole et al., 2011)	
		Stature (Cole et al., 2011)	
		PTA type (Cole et al., 2011)	
		Udder depth (Cole et al., 2011)	
		Teat placement – rear (Cole et al., 2011)	
		Rear leg placement - rear view (Cole et al., 2011)	
		Rear leg placement - side view (Cole et al., 2011)	
		Strength (Cole et al., 2011)	
X:133,244,405, rs29018822	Fat kg (200d LA1)	Body depth (Cole et al., 2011)	Holstein
		PTA type (Cole et al., 2011)	
		Rump width (Cole et al., 2011)	
		Stature (Cole et al., 2011)	
		Udder attachment (Cole et al., 2011)	
		Udder height (Cole et al., 2011)	
		Udder depth (Cole et al., 2011)	
		Strength (Cole et al., 2011)	
		Body depth(Cole et al., 2011)	
